# *Wnt* Signaling Drives Correlated Changes in Facial Morphology and Brain Shape

**DOI:** 10.3389/fcell.2021.644099

**Published:** 2021-03-29

**Authors:** Marta Marchini, Diane Hu, Lucas Lo Vercio, Nathan M. Young, Nils D. Forkert, Benedikt Hallgrímsson, Ralph Marcucio

**Affiliations:** ^1^Department of Cell Biology and Anatomy, University of Calgary, Calgary, AB, Canada; ^2^McCaig Institute for Bone and Joint Health, University of Calgary, Calgary, AB, Canada; ^3^Alberta Children’s Hospital Research Institute, University of Calgary, Calgary, AB, Canada; ^4^Department of Orthopaedic Surgery, University of California, San Francisco, San Francisco, CA, United States; ^5^Department of Radiology, University of Calgary, Calgary, AB, Canada

**Keywords:** *Wnt* signaling, frontonasal ectoderm zone, craniofacial development, geometric morphometric, 3D imaging

## Abstract

Canonical Wnt signaling plays multiple roles critical to normal craniofacial development while its dysregulation is known to be involved in structural birth defects of the face. However, when and how Wnt signaling influences phenotypic variation, including those associated with disease, remains unclear. One potential mechanism is via Wnt signaling’s role in the patterning of an early facial signaling center, the frontonasal ectodermal zone (FEZ), and its subsequent regulation of early facial morphogenesis. For example, Wnt signaling may directly alter the shape and/or magnitude of expression of the *sonic hedgehog* (*SHH*) domain in the FEZ. To test this idea, we used a replication-competent avian sarcoma retrovirus (RCAS) encoding *Wnt3a* to modulate its expression in the facial mesenchyme. We then quantified and compared ontogenetic changes in treated to untreated embryos in the three-dimensional (3D) shape of both the *SHH* expression domain of the FEZ, and the morphology of the facial primordia and brain using iodine-contrast microcomputed tomography imaging and 3D geometric morphometrics (3DGM). We found that increased *Wnt3a* expression in early stages of head development produces correlated variation in shape between both structural and signaling levels of analysis. In addition, altered *Wnt3a* activation disrupted the integration between the forebrain and other neural tube derivatives. These results show that activation of Wnt signaling influences facial shape through its impact on the forebrain and *SHH* expression in the FEZ, and highlights the close relationship between morphogenesis of the forebrain and midface.

## Introduction

Craniofacial development is a highly orchestrated process that involves both physical and molecular interactions between neuroectoderm, mesenchyme, and surface ectoderm. Clinically, the relationship between the brain and the face is well appreciated (DeMyer et al., 1964). The brain helps to physically shape the face and molecular signals from the brain are required for facial development (Marcucio et al., 2015). Recent evidence indicates that signals from neural crest cells contribute to development of the forebrain (Creuzet et al., 2004; Aguiar et al., 2014; Garcez et al., 2014). In this work, we altered signals that participate in development of the face and brain and assess covariance in these structures in order to determine the extent to which development of the brain and face are integrated.

Facial morphogenesis is regulated by cellular interactions that are mediated by various morphogenetic signals, including fibroblast growth factor (FGF), bone morphogenetic protein (BMP), wingless (Wnt), and sonic hedgehog (SHH) signaling (Minoux and Rijli, 2010). Morphogenesis of the upper jaw appears to be regulated by an epithelial signaling center known as the frontonasal ectodermal zone (FEZ). The FEZ is a region of ectoderm spanning the roof of the developing buccal cavity and the dorsal frontonasal process (FNP), defined by strong expression of *SHH* and delimited at the oral boundary of the frontonasal prominence by *FGF8* expression (Hu et al., 2003; Hu and Marcucio, 2009a; Hu et al., 2015). In addition to *SHH* and *FGF8*, other signaling molecules, such as BMPs (Francis-West et al., 1994; Barlow and Francis-West, 1997) and WNTs (Lan et al., 2006; Geetha-Loganathan et al., 2009; Ferretti et al., 2011), are expressed in the FEZ and contribute to FEZ function. However, when *SHH* expression is either absent from the FEZ or when SHH signaling is disrupted in the face, upper jaw morphogenesis is severely compromised (Cordero et al., 2004; Jeong et al., 2004; Hu and Marcucio, 2009b; Kurosaka et al., 2014; Richbourg et al., 2020) indicating that SHH is a key morphogen that mediates FEZ function.

The spatial pattern of *SHH* expression in the FEZ is associated with embryonic facial shape (Hu and Marcucio, 2009a), so that altering the shape of the *SHH* expression domain in the FEZ alters facial morphogenesis. For example, after activating SHH signaling in the brain of chicks, the faces resembled mammalian embryos and the *SHH* expression domain was split into two domains that resembled the mammalian pattern of expression (Hu and Marcucio, 2009b). After transplanting the basal forebrain from duck to chick embryos, facial shape was shifted in morphospace toward the duck and the shape of the *SHH* expression domain correlated with facial shape (Hu et al., 2015). However, what regulates the spatial pattern of *SHH* expression in the FEZ is not known. Conditional inactivation of beta-catenin in the surface ectoderm altered the pattern of *Shh* expression in the mouse FEZ. Normally, this domain is bilateral, and in the absence of canonical Wnt signaling in the FEZ, *Shh* expression appears as a single domain like that in chicks and the embryos had a narrow anteriorly projecting upper jaw that grossly resembled a beak (Reid et al., 2011). Other work in mice also indicates that the pattern of Wnt signal activation in the face is associated with the unique growth patterns in different organisms (Brugmann et al., 2007). Thus, the pattern of morphogenesis of the upper jaw appears related, in part, to the shape of the *SHH* expression domain.

Signaling from the brain to the face is not unidirectional. Recent work has shown that signals from the neural crest cells are required to maintain signaling centers that regulate development of the forebrain (Le Douarin et al., 2012). Neural crest cells maintain the pattern of *WNT*, *SHH*, and *FGF8* expression in the developing forebrain (Creuzet et al., 2004). The effects of the neural crest on forebrain development appear mediated by expression of BMP and WNT antagonists within by neural crest cells (Aguiar et al., 2014; Garcez et al., 2014). Hence, a complex signaling network involving WNT, BMP, and SHH among the brain, facial ectoderm, and neural crest mesenchyme contributes to morphogenesis of the amniote upper jaw and the brain. However, the extent to which these signaling pathways integrate development of these tissues is not known.

The aim of this work was to assess the interactions between the brain and the face by altering Wnt signaling in the developing head. This was achieved by activating the canonical Wnt pathway in the neural crest of developing chick embryos by infecting them with a replication competent virus encoding *Wnt3a* (RCAS-*Wnt3a*; Kengaku et al., 1998). Additionally, we infected the migrating neural crest using RCAS-*Dkk1* (Yue et al., 2006) to inhibit Wnt signaling (Mukhopadhyay et al., 2001). We used 3D geometric morphometrics (3DGM) to quantify the shape of the head and the brain, and we developed four novel metrics based on geometry for quantifying shape and size of a gene expression domain to assess *SHH* expression in the FEZ. We first examined the extent to which activation of Wnt signaling in the developing face regulates the shape of *SHH* expression in the FEZ and then determined whether there is a relationship between the shape of *SHH* expression and the face. Next, we examined how altered Wnt signaling affects the neural tube. Our results show that these metrics of FEZ shape correspond with major axes of craniofacial variation after WNT activation and illustrate the high level of integration between the face and brain.

## Materials and Methods

### Embryo Manipulation

Fertilized chicken eggs (*Gallus gallus*, Charles River, SPAFAS) were prepared for surgical manipulations as follows. Embryos were incubated to Hamburger and Hamilton stage 10 [HH 10 (Hamburger and Hamilton, 1951) (approximately 36 h)] and then a small hole was made in the shell directly over the embryo after removing 1.0 ml of albumin. Sharpened tungsten needles were used to excise the anterior neural folds of embryos. RCAS-*Wnt3a* or RCAS-AP virus injections were carried out using a PV830 Pneumatic Picopump (World Precision Instruments Sarasota, FL, United States). Approximately 100–150 nl of virus solution was injected into the mesenchyme on each side of the forebrain of HH10 embryos. The hole was covered with tape and the embryos were returned to the incubator. Injected embryos were collected at 72 h post treatment. Embryos were removed from the eggs, rinsed in ice-cold PBS, fixed in 4% paraformaldehyde over-night at 4°C, and taken through a graded ethanol series to dehydration. Then prepared for whole mount *in situ* hybridization.

### Preparation of *Wnt3a* Expressing Avian Virus (RCAS-*Wnt3a* and RCAS-*Dkk1*)

Replication-competent avian sarcoma retroviral vector encoding mouse *wnt3a* cDNA (from Addgene cat# CT#169) and *dkk1* cDNA (from Dr. Tingxin Jiang at USC) was produced as described (Fekete and Cepko, 1993). Briefly described, virus stock was prepared by transfection of proviral DNA plasmid into immortalized chicken embryonic fibroblasts (DF-1 cells). Transfected DF-1 cells were expanded in Dulbecco’s Minimum Essential Medium (DMEM) supplemented with 10% fetal bovine serum (FBS). After two passages, cells were grown under low serum conditions (1% FBS) and virion was harvested over three consecutive days from confluent cultures. Viral supernatant was concentrated by centrifugation at 25,000 rpm for 3 h at 4° C. After discarding the supernatants, viral pellets were re-suspended in low serum culture medium, and 10 μl aliquots were frozen at −80 °C.

### Infection With RCAS-*Wnt3a* and RCAS-*Dkk1* Virus

Virus-dye solutions (10 μL of virus supplemented with 1 μl of 0.02% Fast Green) were prepared at the time of injection to allow visualization of injected solutions. A pulled borosilicate glass capillary pipette (OD = 1.0 mm; ID = 0.5 mm; Sutter Instrument, Novato, CA, United States) connected to a KITE-R micromanipulator (World Precision Instruments Sarasota, FL, United States) was loaded with the virus-dye solution. Embryos (SPAFAS) were incubated to HH10 and then a hole was made in the shell. A solution of neutral red was applied to the vitelline membrane to visualize the embryo. Injections were carried out using a PV830 Pneumatic Picopump (World Precision Instruments Sarasota, FL, United States). Approximately 100–150 nl of virus-dye solution was injected into the mesenchyme on each side of the forebrain of HH10 embryos (Supplementary Figure 1A). Viable embryos were euthanized and collected at 48 and 72 h post treatment. To test whether viral infection was similar between treatment groups, we performed whole mount *in situ* hybridization to assess expression patterns of the RCAS viral envelope gene (*v-ENV*; Supplementary Figure 1B). Using *in situ* hybridization, we have previously shown that using this approach, viral infection is restricted to the mesenchyme and ectoderm and is excluded from the neuroepithelium (Foppiano et al., 2007; Supplementary Figure 1C). See below for *in situ* hybridization details.

### Total RNA Preparation and qRT-PCR

Quantitative polymerase chain reaction (qPCR) was performed on tissue containing only mesenchyme and ectoderm (therefore not including the neuroectoderm) dissected from the FNP. Briefly, the FNP was dissected from embryos, digested or 20 min on ice with 2 mg/mL dispase. Neural ectoderm was separated from the surface ectoderm and mesenchyme using tungsten needles and RNA was isolated ectoderm and mesenchyme four normal and four RCAS-Wnt3a infected embryos using RNeasy plus mini kit (Qiagen cat#74134). cDNA synthesis was performed using the Invitrogen Superscript III kit following the manufacturer’s instructions. qRT-PCR was performed using a Bio-Rad CFX96 real-time PCR machine. qRT-PCR primers for specific genes are:

*GAPDH* (Fw: CTGGTATGACAATGAGTTTGG; Rv: ATCAG TTTCTATCAGCCTCTC);

*GAG* (Fw: GGTTGCTTATGTCTCCCTCAG; Rv: GTTGTTT CTCCCACCTCCTC); *WNT3A* (Qiagen, cat#QT00590555)

*AXIN2* (Qiagen, cat#QT01139362). Fold change was calculated based on the 2^–ΔΔCt^ method, and relative quantity was calculated using the 2^–ΔCt^ method. ΔCt was calculated between each target gene and *GAPDH*, a selected housekeeping gene. ΔCt was used for statistical analysis (Supplementary Figure 1D and Supplementary Table 1).

### *In situ* Hybridization

*In situ* hybridization was performed on paraffin-embedded sections or whole embryos as previously described (Albrecht et al., 1997). Briefly, subclones of *v-ENV*, *Shh*, and *Fgf8* were linearized to transcribe DIG-labeled antisense riboprobes or linearized for transcription of ^35^S-labeled antisense riboprobes as previously described (Jeong et al., 2004; Marcucio et al., 2005).

**FIGURE 1:**
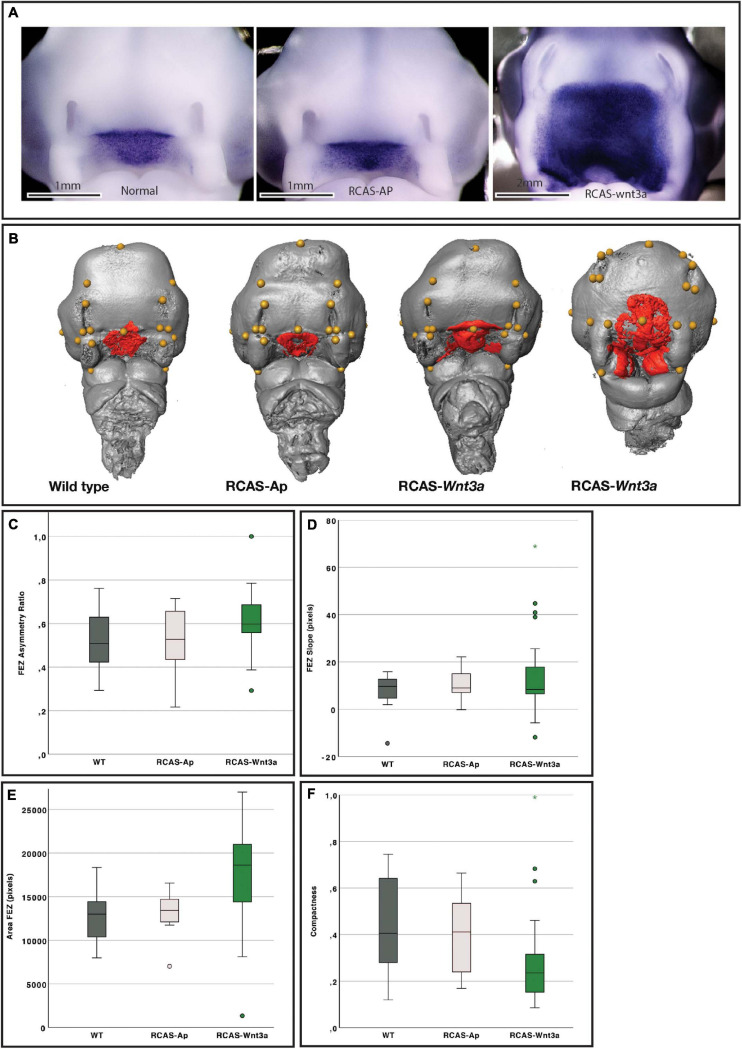
Impact of *Wnt3a* on head and FEZ shape at stage HH22. **(A)** *In situ* hybridization for *SHH*. **(B)** Examples of head (gray) with landmarks (yellow), and FEZ (red) shape identified by Shh expression using *in situ* hybridization. From left to right: wild-type (WT), RCAS-Ap, RCAS-*Wnt3a* with mild phenotype, and RCAS-*Wnt3a* with severe phenotype. Boxplots of FEZ variables: **(C)** asymmetry ratio; **(D)** slope; **(E)** area; **(F)** compactness.

Images of *in situ* hybridization assays are captured using Adobe Photoshop (Figure 1A and Supplementary Figures 2A,B).

### PHH3 Immunohistochemistry and TUNEL Assay

PHH3 IHC was performed with formalin-fixed, paraffin-embedded tissue sections. Briefly described, 8-μm-thick sections were obtained with a microtome, transferred onto adhesive slides, and dried at 37°C overnight. Tissue sections were deparaffinized using xylene and rehydrated. EDTA buffer (pH 9.0) was used for antigen retrieval at 100°C for 10 min. Using 3% H_2_O_2_, endogenous peroxidase was blocked for 10 min at room temperature. Slides were incubated with primary antibodies (PHH3, Polyclonal, 1:200, from Cell Signaling cat#9701S) overnight at 4°C, and a secondary antibody of HRP conjugated anti-rabbit immunoglobulin for 60 min at RT. Samples were incubated in DAD peroxidase substrate for 8–10 min (from Vector Laboratories, Inc., cat#SK-4100). Whole tissue images were acquired, and the number of PHH3 positive cells and total cells were manually and automatically counted in the FNP area. Nuclei were stained by using hoechst 33342 for total cell count. Analyze Particles tool in ImageJ 1.51s (Wayne Rasband, National Institutes of Health, United States) was used to count the cells. We used four embryos for each treatment group (RCAS-Ap and RCAS-Wnt3a) and counted mesenchymal cells in 14 sections in embryos infected with RCAS-Wnt3a and 20 sections for embryos infected with RCAS-Ap. For the neural ectoderm we selected four sections for each treatment group (Supplementary Table 2). We then calculated the mean for each embryo and performed statistical analysis.

For analysis of cell death, embryos were embedded in paraffin, sectioned, and TUNEL staining was performed according to the manufacturer’s protocol (Roche Applied Science, Indianapolis, IN, United States). Sections were counterstained with DAPI to stain the nuclei blue (Supplementary Figure 2C). The slides were mounted with antifluorescence quenching sealing solution and imaged by epifluorescent microscopy using a Leica DMRB microscope.

### 3D Imaging

For FEZ analysis, heads were embedded in 1.5% low melting agarose (Invitrogen) and dehydrated in 100% methanol for 2 days. After this, samples were clarified overnight in BABB (two parts Benzyl Benzoate: one part Benzyl Alcohol). Samples were then imaged twice with optical projected tomography Bioptonics 3000 (Sky Scan, Germany) at 7 μm resolution using two different exposures in the white light range. The first scan had a range of ∼20–30 ms exposure to visualize head shape and the second scan was performed with higher exposure (∼50–70 ms) to visualize shape of the *Shh* expression domain in the FEZ. We used Nrecon software (SkyScan, Germany) to reconstruct the images. Image stacks were then imported into Amira 6 software for visualization. We collected optical projected tomography data for 15 wild-type (WT), 23 RCAS-*Wnt3a*, and 12 RCAS-AP. For head and brain analysis and contrast enhanced microCT, heads were stained with 3.75% Lugol’s iodine for approximately 24 h, embedded in 1% low melting agarose (Invitrogen), and scanned using Scanco μCT35 scanner (Scanco Medical AG, Bruttisellen, Switzerland) with exposure range 55–70 Kv, 89–113 μA, and resolution of 11 μm. After reconstruction, image stacks were imported into Amira software (Version 6, FEI) for visualization and the brain was manually segmented. We collected micro-CT data for five RCAS-AP and 12 RCAS-*Wnt3a* at 48 h and six RCAS-*Dkk1*, six RCAS-AP, and seven RCAS-*Wnt3a* at 72 h.

### Head and Brain Shape Analysis

Using Amira 6 software, one observer (MM) placed 21 landmarks on the surface of the head (Supplementary Figure 3) and an additional 17 landmarks on the surface of the brain (Supplementary Figure 4). Each specimen was landmarked three times whereas the mean coordinates were used for subsequent analyses. We performed general Procrustes alignment, principal component analysis (PCA), and modularity tests using *geomorph 3.0.7* and *Morph* 2.6 packages in Rstudio 1.1.463 (^©^2009–2018 Rstudio, Inc.).

### FEZ Shape Analysis

Sonic hedgehog expression in the FEZ was manually segmented using Amira 6 software. Based on the segmented volumes of the embryo and the FEZ, four metrics were computed to quantify the FEZ shape.

Asymmetry: a bilateral symmetry of the Shh expression can be observed in WT and RCAS-Ap subjects (Figures 1A,B). Furthermore, the symmetry plane of the Shh expression matches the symmetry plane of the embryo, which is used to compute the asymmetry of the FEZ shape assuming that even if the FEZ shape is not symmetric, the embryo itself will still be mostly symmetric. To compute the Shh expression symmetry, a sagittal plane of symmetry is manually defined in the embryo in a first step, and the embryo and segmented Shh expression are mirrored. As this manually defined symmetry plane might not be optimal, the mirrored embryo was registered to the original volume using an intensity-based rigid registration implemented in the open-source Medical Imaging NetCDF (MINC) software (Collins and Evans, 1997; Vincent et al., 2016) to correct slight misplacements between the embryo and the mirrored one. This registration was computed using a multi-resolution approach and optimizing a cross-correlation similarity metric (Devine et al., 2020). After registration, the final rigid transformation is applied to the mirrored Shh expression. This allows to calculate the asymmetry ratio (S) for Shh expression using the matching voxels between the original (F) and mirrored (F_*m*_) Shh expression:

$$S=\left|F-F_{m}\right|/\left|F\right|.$$

Slope (triangularity): The Shh expression in WTs and RCAS-Ap samples typically has a triangular shape (Figures 1A,B). In order to quantify the triangularity, the number of voxels of the segmented FEZ aligned to the embryo sagittal plane were computed for each slice starting from the top to the bottom. After this, the slope resulting from a linear fitting was computed. This slope is negative for triangular-like expressions, while close to zero or positive for other shapes.

Projected area and compactness: The FEZ expression is typically displayed as a irregular and thin surface. Xu et al. (2015) proposed a shape analysis technique that is based on projecting the 3D FEZ expression to a 2D plane. Using this projection, two descriptors were computed: the area of pixels contained in the 2D projected surface, and the compactness C, given by Žunić et al. (2010):

$$C=\frac{4\times \pi \times \text{Area}}{{\text{Perimeter}}^{2}}.$$

### Statistical Analysis

Statistical analyses (Kruskal–Wallis, ANOVA and Spearman’s correlation) were performed in Rstudio 1.1.463 (^©^2009–2018 Rstudio, Inc.) using *dunn.test* package and IBM SPSS 26.0 (Armonk, NY: IBM Corp.). *p*-values below 0.05 were considered statistically significant. Canonical correlation analyses were performed in MATLAB 9.5 (The MathWorks Inc., 2018).

## Results

### Perturbation of *Wnt* Signaling and FEZ Shape

We hypothesized that activation of Wnt signaling in the face would induce changes in expression of *Shh* and, therefore, in changes of the FEZ shape. To activate the Wnt pathway in the face, we injected RCAS-*Wnt3a* (*n* = 23), or RCAS-*alkaline phosphatase* (RCAS-*AP*) as a control (*n* = 12), into the mesenchyme of chick embryos at HH10 (Hamburger and Hamilton, 1951; Supplementary Figure 1A), during neural crest migration into the facial prominences, as previously described (Foppiano et al., 2007). Using this this approach, infection of the mesenchyme and ectoderm occurs but infection of the neural tube is excluded (Supplementary Figure 1C; Foppiano et al., 2007). Embryos were incubated and collected at HH22. We collected 15 additional unmanipulated WT embryos at HH22 as normal controls.

To quantify infection levels, Wnt3a overexpression, and to examine whether infection and expression was restricted to the FNP, we quantified the level of expression of *WNT3A*, *GAG*, and *AXIN2* indicators of Wnt signaling in neural crest cells (Yu et al., 2007) in the mesenchyme and ectoderm together derived from the FNP from four WT and four RCAS-Wnt3a samples (Supplementary Figure 1D). The expression of these genes is significantly higher in embryos infected with RCAS-Wnt3a compared to WT embryos (ANOVA: *WNT3A*, *p*-value<0.001; *GAG*, *p*-value<0.001; *AXIN2*, *p*-value = 0.004).

A wide range of alterations were observed in embryos infected with RCAS-*Wnt3a* (Figures 1A,B). These embryos had larger heads and alterations in *SHH* expression in the FEZ (Figures 1A,B and Supplementary Figures 5A,C,E) while there were no differences in the pattern of *FGF8* expression in the face (Supplementary Figure 2A). In WT and RCAS-AP groups, *SHH* expression in the FEZ was triangular with the apex pointed downward into the stomatodeum, while the FEZ of embryos infected with RCAS-*Wnt3a* displayed a spectrum of shapes. The *SHH* expression domain in embryos infected with RCAS-*Wnt3a* was diffuse and irregularly shaped (Figures 1A,B).

To permit 3D and 2D quantification of shape of the head and *SHH* expression in the FEZ, we imaged all samples, which had been used to assess *SHH* expression via *in situ* hybridization, using optical projection tomography. To investigate whether the shape of the *SHH* domain in the FEZ was different in embryos after activation of Wnt signaling, we calculated the area, asymmetry ratio, slope (indicating whether the FEZ is triangular or square in shape), and compactness (indicating the degree to which the FEZ is circular and compact) of the *SHH* expression domain (see section “Materials and Methods”). Head size, indicated as centroid size, FEZ area, and compactness all differed significantly between WT and RCAS-*Wnt3a* embryos (Kruskal–Wallis test, see Table 1 and Figure 1). *SHH* expression in embryos infected with RCAS-Ap was only significantly different from RCAS-*Wnt3a* in the area of the FEZ (see Table 1). We did not find any difference between treatments using FEZ slope and asymmetry ratio.

**TABLE 1:** Kruskal–Wallis test for the frontonasal ectoderm zone (FEZ) and head size (centroid size).

**Variable**	**WT**	**RCAS-Ap**	**RCAS-Wnt3a**	**Statistic Kruskal–Wallis test**
Sample size	15	12	13	
Centroid size	695.2 (18.2)	724.6 (11.1)	825.6 (28.4)	*T* = 10.736; df = 2; *p*-value = 0.005 Adj. *p*-value: BWT-RCAS-Ap = 1 **WT-RCAS-Wnt3a = 0.0023** RCAS-Ap-Wnt3a = 0.0774
FEZ asymmetry	0.517 (0.039)	0.518 (0.046)	0.618 (0.0	*T* = 4.911; df = 2; *p*-value = 0.086
FEZ slope	7.69 (1.96)	10.39 (1.82)	15.25 (3.79)	*T* = 1.233; df = 2; *p*-value = 0.54
FEZ area	**12,483 (795)**	**13,191 (707)**	**17,297 (1174)**	*T* = 12.851; df = 2; *p*-value = 0.002 Adj. *p*-value: BWT-RCAS-Ap = 1 **WT-RCAS-Wnt3a = 0.0015** **RCAS-Ap-Wnt3a = 0.0166**
FEZ compactness	**0.433 (0.055)**	0.396 (0.047)	**0.292 (0.045)**	*T* = 7.030; df = 2; *p*-value = 0.03 Adj. *p*-value: WT-RCAS-Ap = 1 **WT-RCAS-Wnt3a = 0.0249** RCAS-Ap-Wnt3a = 0.079

*Columns 2–4 indicate means with standard error presented in parenthesis. Last column indicates the test statistics. *T* indicates test statistic. df indicates degree of freedom. *p*-value indicates the asymptotic significance (two-sided test). Adj. *p*-value indicates asymptotic significances (two-sided tests) adjusted by Bonferroni correction. Terms in bold indicate significance.*

**FIGURE 2:**
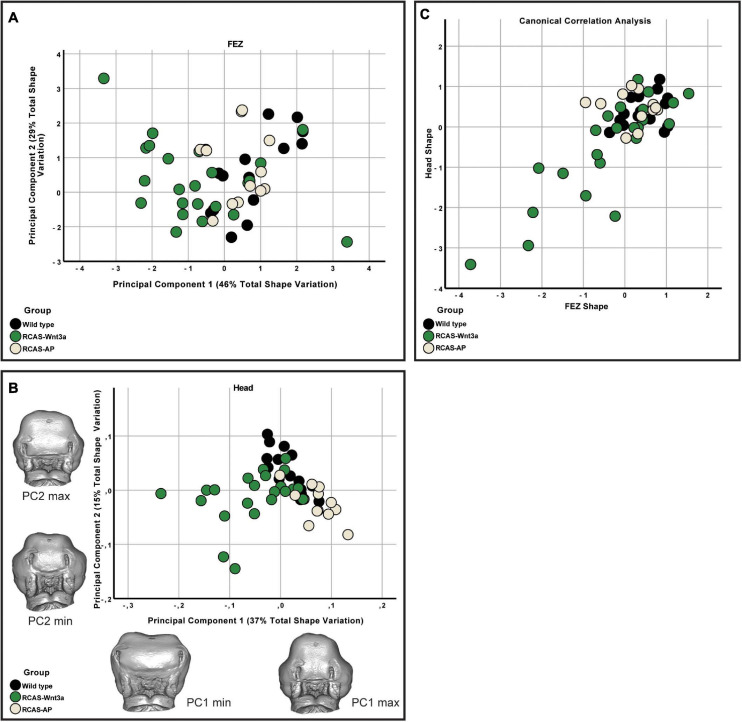
FEZ and head shape variation between wild-type, RCAS-Ap and RCAS-*Wnt3a* at stage 22/23. **(A)** Biplot of first two principal components of FEZ shape based on four variables. **(B)** Biplot of first two principal components of head shape based on 21 landmarks. Gray meshes represent head shape at the extreme of the principal component. **(C)** Canonical correlation analysis of the first five principal components of the head and the four principal components of the FEZ.

We expected some degree of covariation in these metrics as changes in FEZ shape and size will produce concerted changes in all these measures. To identify the primary axis of this covariation, we performed PCA of the four FEZ shape variables. Of the four principal components PC1 and PC2 represent 46 and 29% of the total variation, respectively (Figure 2A). FEZ shape differs significantly between the groups (Kruskal–Wallis: *T* = 15.73, df = 2, *p*-value < 0.01). We found no evidence that craniofacial variation is due to embryonic manipulation alone, as WT and RCAS-Ap are not significantly different (Bonferroni correction *p*-value = 1). However, both, WT (Bonferroni correction *p*-value = 0.0007) and RCAS-Ap (Bonferroni correction *p*-value = 0.0036), differ significantly from RCAS-*Wnt3a*, suggesting that activation of Wnt signaling in the face produces significant differences in FEZ shape by expanding the domain of *SHH* expression.

### Head and FEZ Shape Relationship

Given that the shape of *SHH* expression in the FEZ is associated with facial shape, we wanted to assess the extent to which the quantified differences in the shape of the FEZ might correspond to differences in head shape. Therefore, we analyzed the head shapes using 3DGM. Using Amira 6, we placed 21 landmarks on images obtained using micro-CT imaging. The landmarks represent a subset of the set used in Smith et al. (2015; Supplementary Figure 3). The resulting landmark sets were aligned using a general Procrustes analysis and major axes of covariation were extracted using PCA. The first five principal components account for approximately 72% of the total variance in head shape. From PC6 onward, variation along each principal component explains no more than 5% of the total covariance. As these components represent such a small aspect of the total overall variance in head shape, we limited subsequent analyses of head shape to PC1 through PC5, with PC1 and PC2 representing 37 and 15% of the total variation, respectively (Figure 2B). We found significant differences in head shape between the groups (Kruskal–Wallis: *T* = 21.5896, df = 2, *p*-value < 0.01). As with the FEZ, we found that retroviral infection alone did not produce differences in head shape in comparison with WT embryos (*p*-values). However, after activating Wnt signaling in embryos, significant differences in head shape were observed (Bonferroni correction, WT *p*-value < 0.0001, RCAS-Ap = 0.001) and were primarily along the first principal component (Figure 2). We conclude that increased mesenchymal expression of RCAS-*Wnt3a* is sufficient to drive changes in embryo head shape, particularly wider faces, and differences in the maxillary processes (Figures 1, 2).

We then investigated whether overall continuous variation in the shape of *SHH* expression in the FEZ could be correlated with overall continuous variation in head shape forming a single morphocline. To identify axes of covariation shared between FEZ shape and head shape, we used canonical correlation analysis following the methodology described by Hu et al. (2015). Here, we found that variation in head and FEZ shape is relatively limited in both WT and RCAS-Ap embryos, whereas both head and FEZ shape are much more variable after activating Wnt signaling in embryos. This variation is highly correlated (Spearman’s correlation, *R* = 0.559, *p*-value < 0.001) with the most extreme FEZ shapes corresponding with the most extreme head shapes. Hence, activation of Wnt signaling drives correlated changes in the shape of *SHH* expression in the FEZ and overall head shape.

### Head and Brain Shape Relationship

**FIGURE 3:**
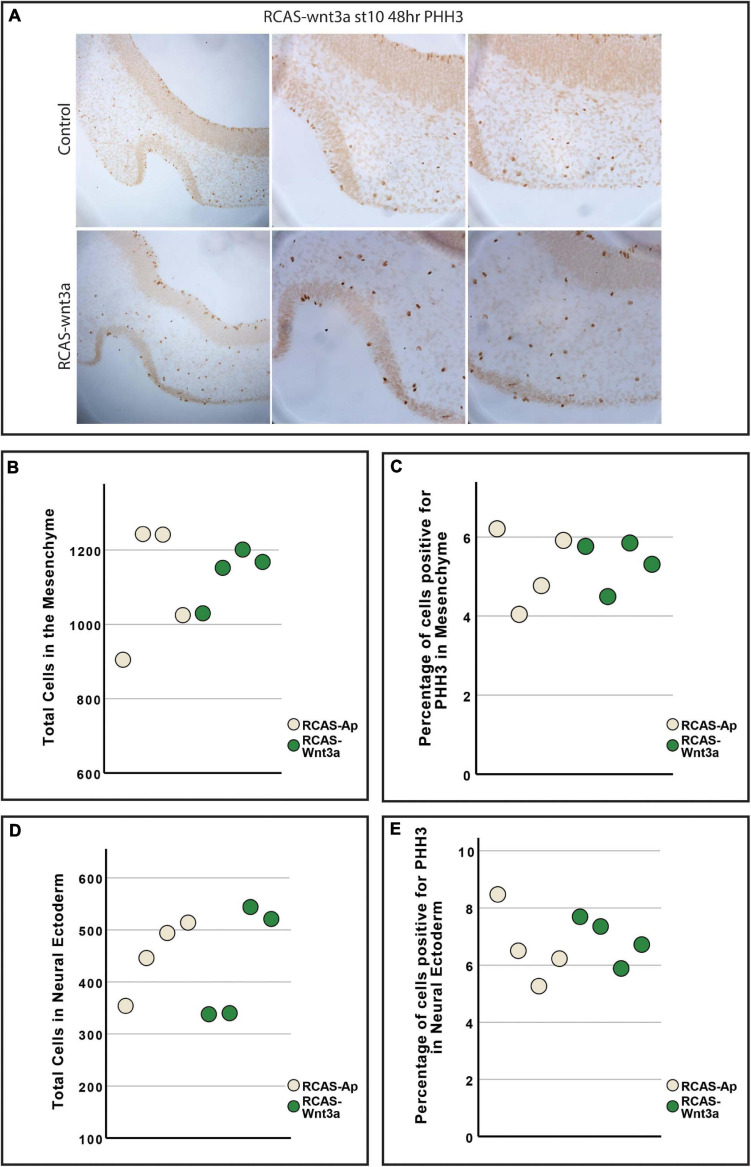
Cell proliferation is unchanged between RCAS-Ap and RCAS-*Wnt3a* at stage HH22. **(A)** Immunohistochemistry for phosphohistone H3 (PHH3). Boxplot of total cells **(B)** and percentage of cells positive for PHH3 **(C)** in mesenchyme. Boxplot of total cells **(D)** and percentage of cells positive for PHH3 **(E)** in neural ectoderm (bottom).

Previous studies have shown that *Wnt3a* activation in the chicken face leads to increased cell proliferation and mesenchymal tissue (Brugmann et al., 2010), while inhibition of *Wnt3a* in mice disrupts proper neural development (Augustine et al., 1993). Thus, we investigated next whether our observed changes in facial morphology could be explained by differences in *SHH* expression, proliferation or apoptosis in the face and brain. To test whether Wnt3a affects *SHH* expression in the neural ectoderm, we performed *in situ* hybridization for *SHH* in head sections (Supplementary Figure 2B). Our *in situ* hybridization shows lateral and dorsal expansion of *SHH* expression in the neural ectoderm, suggesting a similar relationship between Wnt3a signaling and *SHH* expression as seen in the FEZ (Figure 1A and Supplementary Figure 2B). To quantify proliferation, we performed anti-phosphohistone-H3 immunostaining and quantified number of total and proliferating cells in the mesenchyme and in the neural ectoderm (Kim et al., 2017; Figure 3). We did not observe an increase in either total tissue (ANOVA: *p*-value = 0.722; Figure 3B) or proliferating cells (ANOVA: *p*-value = 0.846; Figure 3C) in the mesenchyme. We also did not observe an increase in either the number of cells (ANOVA: *p*-value = 0.815; Figure 3D), or of proliferating cells (ANOVA: *p*-value = 0.929; Figure 3E) in the neural ectoderm. To qualitatively assess if there was an increase in apoptotic cells, we performed TUNEL assay (Supplementary Figure 2C). We did not observe any difference in apoptotic cells in the neural ectoderm or mesenchyme.

Since we observed an increase in head size (Figures 1A,B and Supplementary Figure 5) after activating Wnt signaling, we investigated whether the early effect of *Wnt3a* in shaping the head may be achieved through morphogenesis of the brain rather than mesenchymal proliferation which was examined in previous research on later stages of facial development (Brugmann et al., 2010). Therefore, we analyzed whether overall shape and size of the brain was affected by activation of Wnt signaling in the face. We used contrast enhanced microCT to visualize the 3D shape of the brain and head of embryos infected with RCAS-*AP* and RCAS-*Wnt3a* after 48 (∼HH18) and 72 h (∼HH22) of treatment. We manually segmented the brain from microCT stacks, and placed 17 landmarks on the brain using Amira 6 software and then quantified shape differences using 3DGM analysis (Supplementary Figure 4). We also placed 21 facial landmarks on the surface of the head to quantify head shape variation (Supplementary Figure 3).

**FIGURE 4:**
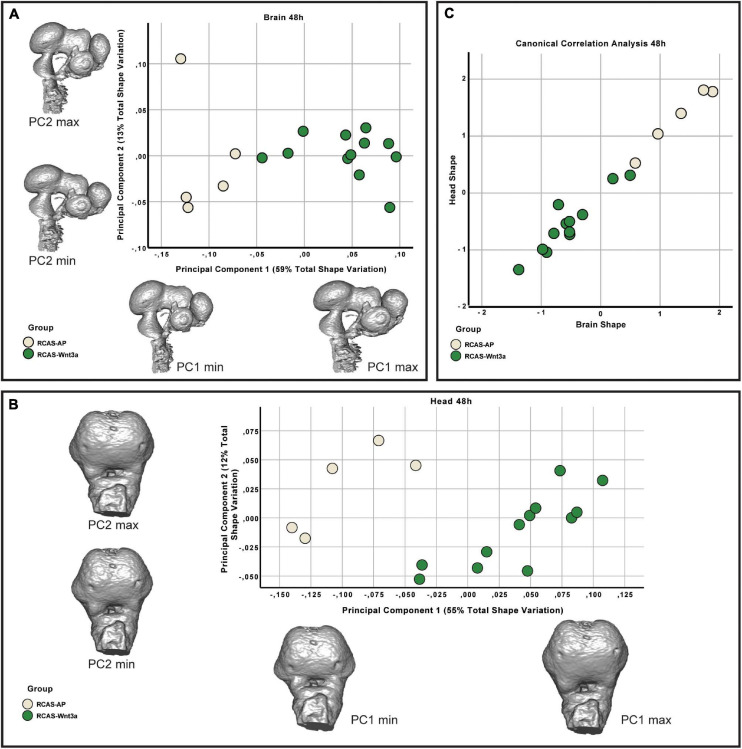
Brain and head shape variation between RCAS-Ap and RCAS-*Wnt3a* stage HH18. **(A)** Biplot of the first two principal components of the brain based on 17 landmarks. Gray meshes represent brain shape at the extreme of the principal component. **(B)** Biplot of the first two principal components of the head based on 21 landmarks. Gray meshes represent head shape at the extreme of the principal component. **(C)** Canonical correlation analysis of the first five principal components of the head and the first four principal components of the brain.

The resulting landmark sets were aligned using a general Procrustes analysis and major axes of covariation were extracted using PCA. At 48 h post-treatment, the first four principal components account for approximately 85% of the total variance in brain shape and, similarly, the first five principal components account for approximately 86% of the total variance in head shape. Respectively, from PC5 and PC6 onward, variation along each principal component explains no more than 5% of the total shape variation. As these components represent such a small aspect of the total overall variance in brain and head shape, we limited subsequent analyses of shape variation only to the PCs that explain more than 5% of total shape variation. In doing so, we found significant differences in brain (Kruskal–Wallis: *T* = 6.4, df = 1, *p*-value = 0.01) and head (Kruskal–Wallis: *T* = 4.0111, df = 1, *p*-value = 0.05) shape between the RCAS-*Wnt3a* and RCAS-Ap groups. We conclude that increased mesenchymal expression of RCAS-*Wnt3a* is sufficient to drive changes in embryo brain and head shape as early as 48 h after treatment with RCAS-Wnt3a, producing a wider face, higher nasal prominence, and differences in the maxillary process (Figure 4).

**FIGURE 5:**
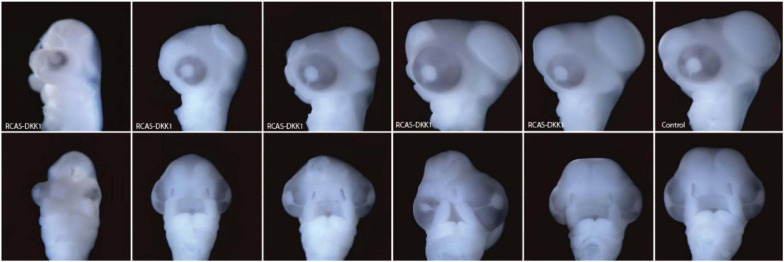
Range of phenotypes observed in RCAS-*Dkk1* at HH22. Top row: lateral view. Bottom row: anterior view. Typical control on far right.

To investigate brain development at 72 h post-treatment, we infected the neural crest of chick embryos with RCAS-AP, RCAS-*Wnt3a*, and RCAS-Dkk1 at HH10 as above. DKK1 is a *Wnt* inhibitor essential for proper head development (Mukhopadhyay et al., 2001). We hypothesized that blocking Wnt signaling in the mesenchyme by infecting the neural crest with RCAS-*Dkk1* should produce a phenotype that appears opposite to embryos infected with RCAS-*Wnt3a* treatment, such as smaller heads and brains. We observed this relatively mild phenotype in most RCAS-*Dkk1* treated embryos (Figure 5).

**FIGURE 6:**
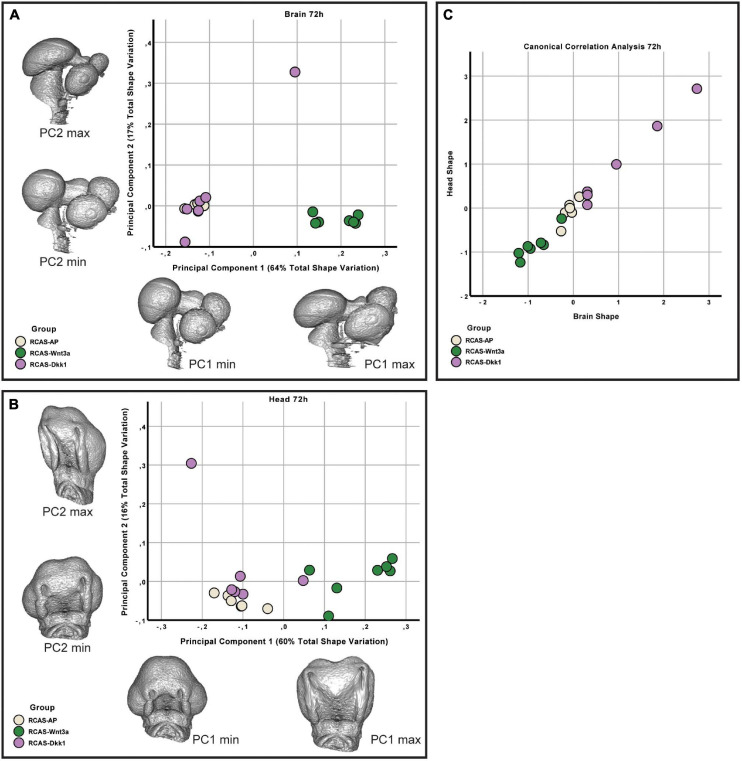
Brain and head shape variation between RCAS-Ap, RCAS-*Dkk1* and RCAS-*Wnt3a* at stage HH22. **(A)** Biplot of the first two principal components of the brain based on 17 landmarks. Gray meshes represent brain shape at the extreme of the principal component. **(B)** Biplot of the first two principal components of the head based on 21 landmarks. Gray meshes represent head shape at the extreme of the principal component. **(C)** Canonical correlation analysis of the first three principal components of the head and of the brain.

At HH22, the sum of the first three principal components explains approximately 87 and 90% of the total variance in head and brain shape, respectively. From PC3 onward, variation along each principal component explains less than 5% of the total shape variation. Therefore, only the first three PCs were considered in this analysis. We found significant differences in head shape only between RCAS-*Wnt3a* and RCAS-Ap treatments (Kruskal–Wallis: *T* = 12.4677, df = 2, *p*-value < 0.01; Bonferroni correction: *p*-value = 0.0006) and not between RCAS-*Dkk1* and the other two groups: RCAS-Ap (Bonferroni correction: *p*-value = 0.0603) and RCAS-*Wnt3a* (Bonferroni correction: *p*-value = 0.2460). In brain shape, we found statistical differences between RCAS-*Wnt3a* and the other two groups (Kruskal–Wallis: *T* = 8.7737, df = 2, *p*-value = 0.01) RCAS-AP (Bonferroni correction: *p*-value = 0.0215) and RCAS-*Dkk1* (Bonferroni correction: *p*-value = 0.0136), while there were no differences between RCAS-*Dkk1* and RCAS-*Wnt3a* (Bonferroni correction: *p*-value = 1) (Figure 6).

We then investigated whether overall continuous variation in brain shape is correlated with overall continuous variation in head shape forming a single morphocline. As with our comparison of head and FEZ shape, we used canonical correlation analysis. Here, we found that variation in brain and head shape is highly correlated both at 48 h (Spearman’s correlation, *R* = 0.929, *p*-value < 0.001) and at 72 h (Spearman’s correlation, *R* = 0.979, *p*-value < 0.001) (Figure 6). This variation formed a single morphocline, with RCAS-*Dkk1* on one end and RCAS-*Wnt3a* on the other, with RCAS-AP distributed between the two.

We then analyzed whether the effect of RCAS-*Wnt3a* treatment was restricted to the forebrain regions adjacent to the injection site, or whether the effects are more broadly experienced by the developing brain. Therefore, we tested whether shape variation in the forebrain is integrated with the hindbrain/midbrain using a modularity test that calculates a covariance ratio (CR) for *a priori* modules (here forebrain versus hindbrain/midbrain), which is then compared to the distribution of CRs for randomly assigned modules across 1000 bootstrap replicates. We found that shape variation in the forebrain was covaried strongly with variation in the hindbrain/midbrain at 48 h post-treatment in the entire sample (CR = 1.033, *p*-value = 0.104) as well as within each treatment group (RCAS-AP: CR = 0.9864, *p*-value = 0.127; RCAS-*Wnt3a*: CR = 1.0647, *p*-value = 0.688). However, by 72 h post-treatment, the strength of covariation between forebrain and hindbrain/midbrain is significantly reduced in the overall sample (CR = 1.0051, *p*-value = 0.02). This decreased integration between forebrain and hindbrain/midbrain is driven entirely by the RCAS-*Wnt3a* group (CR = 0.9275, *p*-value = 0.003), whereas in RCAS-AP (CR = 1.0196, *p*-value = 0.365) and RCAS-*Dkk1* (CR of 1.0368, *p*-value = 0.264), this integration is maintained. Therefore, we conclude that activation of Wnt signaling produces novel variation and covariation within the forebrain alone, and this occurred in the window between 48 and 72 h post-infection.

**FIGURE 7:**
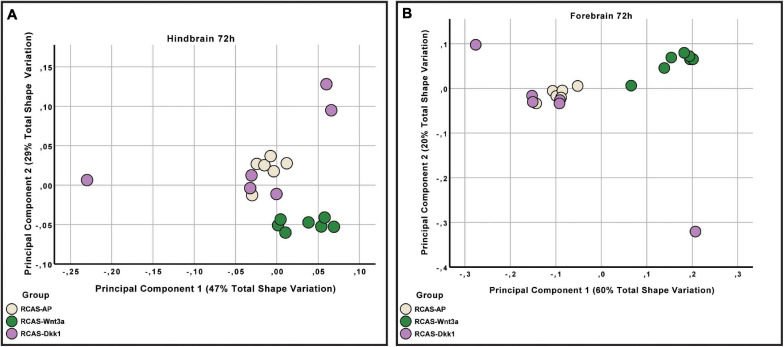
Hindbrain and forebrain shape variation between RCAS-Ap, RCAS-*Dkk1* and RCAS-*Wnt3a* at stage HH22. **(A)** Biplot of the first two principal components of the hindbrain based on six landmarks. **(B)** Biplot of the first two principal components of the forebrain based on 11 landmarks.

As covariation structure between the forebrain and hindbrain/midbrain appears to be disrupted after activation of Wnt signaling, we investigated how shape variation in each anatomical region differed as a result of each treatment (RCAS-AP, RCAS-*Wnt3a*, and RCAS-*Dkk1*). In order to compare shape variation within the forebrain and hindbrain/midbrain, we performed separate analyses of landmark sets representing each region. For both regions, only the first three PCs explained more than 5% of the total variation each so that subsequent analyses were restricted to these PCs. We did not find significant differences in the shape of the posterior part of the brain between treatment groups (Kruskal–Wallis: *T* = 5.2308, df = 2, *p*-value = 0.07), but, as expected, we found significant differences in forebrain shape between embryos infected with RCAS-*Wnt3*a and the two other groups (RCAS-*AP* and RCAS-*Dkk1*) (Kruskal–Wallis: *T* = 11.4391, df = 2, *p*-value < 0.01; Bonferroni correction for RCAS-AP and RCAS-*Wnt3a p*-value = 0.0049; for RCAS-*Dkk1* and RCAS-*Wnt3a p*-value = 0.0068) (Figure 7). We conclude that mesenchymal activation of canonical *Wnt* signaling drives changes in brain and head shape, primarily by driving increased size of the forebrain adjacent to *Wnt3a*-expressing mesenchyme. Inhibiting Wnt signaling with *Dkk1* appears to have a weak opposite effect, but with high variability in the observed phenotype.

## Discussion

Clinically, the relationship between the developing brain and face is well recognized. Patients with holoprosencephaly have coordinated alterations in the brain and face (DeMyer et al., 1964; DeMyer, 1975; Patterson, 2002; Plawner et al., 2002; El-Hawrani et al., 2006). Signals from the brain play a critical role in regulating the morphogenesis of the face, and disruptions to these signals lead to alterations in the brain and in the face (Marcucio et al., 2005; Hu and Marcucio, 2009b; Hu et al., 2015; Geoghegan et al., 2017; Richbourg et al., 2020). In this work, we show that integration of the brain and face is disrupted after activation of Wnt signaling in the head by misexpression of *Wnt3a* in the neural crest mesenchyme. We observed changes in the shape of the *SHH* expression domain in the FEZ that were associated with the severity of the phenotypic outcome, while the correlated changes in the shape of the forebrain and the face illustrate that shared molecular signals between the brain and face coordinate development of this region of the embryo.

### Role of Wnt in Normal and Abnormal Facial Development and Integration With the Brain

Wnt signaling has many distinct and overlapping roles in craniofacial development, which have been studied in multiple contexts. The importance of Wnt signaling in head specification and craniofacial development was originally identified in Xenopus where Wnts and Wnt antagonists were found to have critical roles in specifying the head and central nervous system (McMahon and Moon, 1989; Sokol et al., 1991). Subsequent studies in chicken and mouse have further expanded our understanding of the role of these pathways in craniofacial development. Expression of multiple Wnt ligands, receptors, and effectors have been observed throughout various regions of the developing head of mouse and chick embryos (Lan et al., 2006; Geetha-Loganathan et al., 2009; Vendrell et al., 2009; Paiva et al., 2010). In the mouse, active Wnt signaling is observed in the forebrain and distal parts of the FNP during early stages of outgrowth (Lan et al., 2006; Mani et al., 2010), and our previous work in chick embryos demonstrates active WNT expression throughout the maxillary processes and FNP during outgrowth of these regions (Hu and Marcucio, 2009b). WNT ligands, receptors, and effectors have also been shown to be active in later phases of craniofacial development, including differentiation of skeletal tissues (Liu et al., 2008a) and specification of the retina (Fujimura et al., 2009) and tooth buds (Liu et al., 2008b).

Much of this research has focused on the role of Wnt signaling in regulating the outgrowth of the facial prominences. Blocking or activating Wnt signaling in avian embryos led to malformations of the upper jaw (Shimomura et al., 2019). Activating Wnt signaling in avian embryos using a bead soaked in WNT3a or a retrovirus to misexpress *Wnt3a* beginning at stage 20 led to malformations of the face due to increased cell proliferation in the mesenchyme and increased expression of *BMP2* and *BMP4* (Brugmann et al., 2010), which are targets of FEZ activity (Hu and Marcucio, 2009a), and removing combinations of *Tcf4* and *Lef1* from mice disrupted facial morphogenesis (Brugmann et al., 2007). Using conditional approaches, Wnt signaling was shown to be required within the facial ectoderm and forebrain for development of the FNP derivatives, while activating Wnt signaling throughout the facial ectoderm and forebrain led to facial dysmorphology (Wang et al., 2011). However, more specific approaches, in which beta-catenin was removed from the facial ectoderm, led to alterations in the shape of the developing head associated with alterations in *Shh* expression in the mouse (Reid et al., 2011).

Wnt signaling is also understood to have a later role in formation and fusion of the primary palate (reviewed in: He and Chen, 2012). Multiple *WNT* genes have been identified as risk variants for cleft lip in humans (Chiquet et al., 2008), and knockout of *Wnt9b* in mice leads to cleft lip (Lan et al., 2006) in part by reducing proliferation of the mesenchyme comprising the midface and preventing apposition of the facial primordia (Jin et al., 2012). Furthermore, knock-out of *Wnt9b* and *Rspondin* synergize to produce a more severe bilateral cleft lip (Jin et al., 2020). Additionally, the A/Wsyn mouse, which also exhibits partial penetrance of cleft lip, appears to be caused by changes in methylation in genomic regions adjacent to *Wnt9b* (Juriloff, 1982; Ciriani and Diewert, 1986; Forbes et al., 1989; Green et al., 2019). Palate fusion interacts dynamically with face shape more generally. A phylotypic stage of development among amniotes occurs during fusion of the primary palate, suggesting that facial shape is an important contributor to successful fusion of the primary palate (Young et al., 2014).

Our results show that activation of *Wnt3a* in the mesenchyme beginning at an early time point in craniofacial development (HH10) has a substantial effect on brain development as early as 48 h after treatment, inducing covarying changes in brain shape and size (Figure 4) and expanded *SHH* expression domain in the neural ectoderm along the ventral portion of the neural tube (Supplementary Figure 2B), with additional later effects primarily restricted to the forebrain. Along with the alteration in the covariation of forebrain and midbrain, these results strongly suggest that forebrain morphogenesis is altered in response to modulating activation of Wnt signaling. This contrasts with the lack of observable differences in proliferation in the facial prominences and suggests that mesenchyme-forebrain signaling may be a primary avenue by which Wnt signaling controls facial shape during these early stages of craniofacial development. This early role for *Wnt* signaling contrasts with an identified later role of *Wnt* signaling in driving proliferation within the facial prominences (Brugmann et al., 2010). Our findings are consistent with previous work identifying an important role for *Wnt3a* in early forebrain development and expansion in mouse (Augustine et al., 1993). We can suggest here that this may be achieved by expanding the expression domain of *SHH* in both the FEZ and ventral neural tube. This relationship between activation of Wnt signaling and neural expansion are facilitated by the close spatial relationship of these tissues at HH10, as WNTs are a class of diffusible proteins (Brafman and Willert, 2017). This raises questions of whether mesenchymal Wnt signaling may play a role in initial patterning of the dorsoventral axis of the neural tube, which is achieved largely by SHH signaling.

Interestingly, alterations in the shape of the brain are observed in patients with cleft lip and palate (Kjaer, 1995; Nopoulos et al., 2007; Chollet et al., 2014). Using multiple strains of WT mice, we investigated developmental integration of the brain and face. The variation in the shape of the forebrain was correlated with shape of the face. As the forebrain grows it stretches the facial prominences. Thus, if the rate of growth of the brain is not integrated with growth of the face, the apposition of the facial prominences is altered and this may lead to orofacial clefts (Parsons et al., 2011). In our current work, we were unable to examine primary palate formation due to poor survival to these later stages, but our results may begin to provide unique insights into mechanisms by which the brain and face are physically integrated through shared molecular signals. This may be mediated by neural crest cells, which modulate Wnt signaling to maintain signaling centers that control forebrain development (Aguiar et al., 2014), and as outlined above, also respond to Wnt signals to control growth of the facial primordia and fusion of the primary palate.

### Role of Wnt Signaling in Formation of the FEZ

Sonic hedgehog expression in the FEZ is essential for morphogenesis of the midface (Cordero et al., 2004; Lipinski et al., 2010), but the mechanisms that control its expression are unknown. Our previous work has focused on the role of the forebrain and neural crest cells in regulating *Shh* expression (Marcucio et al., 2005; Hu and Marcucio, 2009b, 2012; Chong et al., 2012; Hu et al., 2015; Richbourg et al., 2020). This work has revealed that Shh signaling from the brain is required during early stages of facial development (Chong et al., 2012), and later BMP dependent signaling within the neural crest also participates in regulating *SHH* expression in the FEZ (Foppiano et al., 2007). Here we observed significant changes in *SHH* expression in the FEZ that appeared to be an expansion of the domain. This suggests that Wnt signaling participates in induction, maintenance or expansion of *SHH* expression in the FEZ.

While the gene regulatory network that controls *Shh* expression is not known, a direct connection between canonical *Wnt* signaling and FEZ specification has been shown (Reid et al., 2011). Gain of function and loss of function experiments targeting β-Catenin in mice have shown that the lateral extent of epithelial *Shh* expression in the FEZ depends on dosage of activated β-Catenin. Loss of function restricted epithelial *Shh* expression medially and gain of function resulted in more lateral epithelial *Shh* expression domains (Reid et al., 2011). Interestingly, we found more lateral expression of *SHH* in embryos after activation of Wnt signaling, with expression extending into the maxillary processes. However, we do not find the same medial exclusion of *SHH* expression seen in the mouse model. This may reflect differences in facial patterning and palatal construction between mouse and chicken rather than a conflict between the results. For example, neural crest cells appear required for *SHH* expression in the FEZ (Kjaer, 1995), but the midline of the mouse face is comprised of a furrow and there are no neural crest cells present until the two median nasal processes merge at the midline (Hu and Marcucio, 2009b). Alternatively, or in combination with direct regulation of *SHH* expression by WNTs in both neural ectoderm and FEZ, other possibilities exist for expanding the *SHH* expression domain. *Wnt3a* may be reducing expression of an inhibitory influence on *SHH* expression, although the identity of such an inhibitor is purely speculative. An additional possibility is that the facial ectoderm is specified normally, but as the forebrain expands the FEZ stretches to accommodate the brain. These are not mutually exclusive scenarios and distinguishing among them is challenging.

### Wnt Signaling in Craniofacial Development and the Palimpsest Model

We show here that early perturbation to *Wnt3a* expression in the facial mesenchyme is sufficient to drive changes in the shape of the FEZ signaling center as well as the shape and size of the forebrain. These changes are distinct from the effects of later perturbations to *Wnt3a* expression in the facial mesenchyme, which primarily drive changes in mesenchymal proliferation (Brugmann et al., 2010). Our experimental approach is unable to determine whether induced changes in the FEZ and forebrain have a causal relationship with each other. We have not determined whether the size of the FEZ drives neural tube cavitation or if these represent parallel responses to the mesenchymal signal. However, these changes in the brain, face, and FEZ shape are strongly covarying and are also distinct from those induced by the same treatment at later stages in development.

These results are consistent with the palimpsest model for morphological integration (Hallgrímsson et al., 2009). In this model, developmental processes drive covariation among phenotypic traits, but these processes act at different times, with later acting processes often obscuring or modulating the effects of those that occur earlier. Here, *Wnt3a* drives a covariation in the brain, face, and FEZ shape during early face formation, while at later stages, variation in the brain and face are decoupled, with *Wnt3a* activation driving covariation among facial components but not the brain. Quantitative approaches, such as our approach here, have shown that craniofacial morphology is highly integrated. This applies to the constellations of features that characterize craniofacial anomalies and facial shape effects of major mutations but also to standing variation within naturally occurring populations (Hallgrímsson et al., 2009, 2020; Martinez-Abadias et al., 2012; Cole et al., 2017). This is important because these patterns of covariation reveal underlying regularities in the relationship between developmental mechanisms and phenotypic variation. In this work, we have shown how variation in the activation of the same gene results in different patterns of covariation over the course of craniofacial development. Understanding the spatiotemporal dynamics of the generation of continuous variation by development is key to unraveling the complex relationships between genetic and phenotypic variation.

## Data Availability Statement

The original contributions presented in the study are included in the article/Supplementary Material, further inquiries can be directed to the corresponding author/s.

## Ethics Statement

Ethical review and approval was not required for the animal study because the study was conducted entirely on chicken embryos prior to hatching. The NIH Office of Laboratory Animal Welfare interprets the Public Health Service Policy on Humane Care and Use of Laboratory Animals as applicable to chicken offspring only after hatching. Therefore, these embryos are exempt from IACUC approval.

## Author Contributions

RM, NMY, and BH designed and conceived the study. MM and DH performed the experiments. MM and DH collected and analyzed the data with assistance of LLV and NDF. MM, RM, and BH wrote the manuscript. All authors provided comments, read, and approved the final manuscript.

## Conflict of Interest

The authors declare that the research was conducted in the absence of any commercial or financial relationships that could be construed as a potential conflict of interest.

## Supplementary Material

Supplementary Figure 1 Experimental procedure and validation of Wnt3a overexpression. **(A)** Picture of a St10 chicken indicating the sites of RCAS injection (red arrows). **(B)** Whole mount *in situ* hybridization for *v-ENV.* **(C)** *In situ* hybridization in section for *v-ENV* in red showing that the RCAS infection is mesenchyme specific, blue nuclear staining. **(D)** Boxplot representing differences -delta CT for *WNT3A*, *AXIN2*, and *GAG* expression.

Supplementary Figure 2 Ectopic expression of Wnt3a affects Shh expression but not FGF8 and apoptosis. **(A)** Whole embryo *in situ* hybridization for *FGF8*. **(B)** *In situ* hybridization for Shh in head section. **(C)** TUNEL assay in head section in green cell death in blue nuclear staining.

Supplementary Figure 3 Landmarks used to quantify shape of the head. Surface render of the head in gray and 21 landmarks in yellow in anterior **(A)**, lateral **(B)**, and dorsal **(C)** view.

Supplementary Figure 4 Landmarks used to quantify shape of the brain. Surface render of the brain in purple and 17 landmarks in yellow (forebrain) and red (mid-hindbrain) in ventral **(A)**, dorsal **(B)**, lateral **(C)**, and posterior oblique **(D)** view.

Supplementary Figure 5 Comparison of centroid size across experiments. **(A)** Centroid size of chicken head at HH22 used for the FEZ analysis. **(B)** Centroid size of the brain of chickens at HH18 based on 17 landmarks. **(C)** Centroid size of the head of chickens at HH18 based on 21 landmarks. **(D)** Centroid size of the brain of chickens at HH22 based on 17 landmarks. **(E)** Centroid size of the head of chickens at HH22 based on 21 landmarks.

Supplementary Table 1 qPCR data of *GAPDH*, *WNT3A*, *AXIN2*, and *GAG* expression in wild-types and RCAS-Wnt3a.

Supplementary Table 2 Quantification of total cells and PHH3 positive cells in mesenchyme and neural ectoderm in sections.

Supplementary Table 3 Raw data of FEZ variables.

Supplementary Table 4 File containing classifier variables for 48hr treatments used in R code called “group_48”.

Supplementary Table 5 File containing classifier variables for 72hr treatments used in R code called “group72-BH”.

Supplementary Table 6 Landmark coordinates for brain shape at 48hr treatment. File used in R code called “landmarks_48_BR”.

Supplementary Table 7 Landmark coordinates for head shape at 48hr treatment. File used in R code called “landmarks_48H”.

Supplementary Table 8 Landmark coordinates for brain shape at 72hr treatment. File used in R code called “landmarks_72B”.

Supplementary Table 9 Landmark coordinates for brain shape at 72hr treatment. File used in R code called “landmarks_72H”.

Supplementary Table 10 Landmark coordinates for head shape. File used in R code called “landmarksW”.

Supplementary Table 11 R script used in analyses.
