# Supplementary figures and images for: Wnt Signaling Drives Correlated Changes in Facial Morphology and Brain Shape
Source: Front Cell Dev Biol. 2021 Mar 29;9:644099. doi: 10.3389/fcell.2021.644099 (PMC8039397; doi:10.3389/fcell.2021.644099)

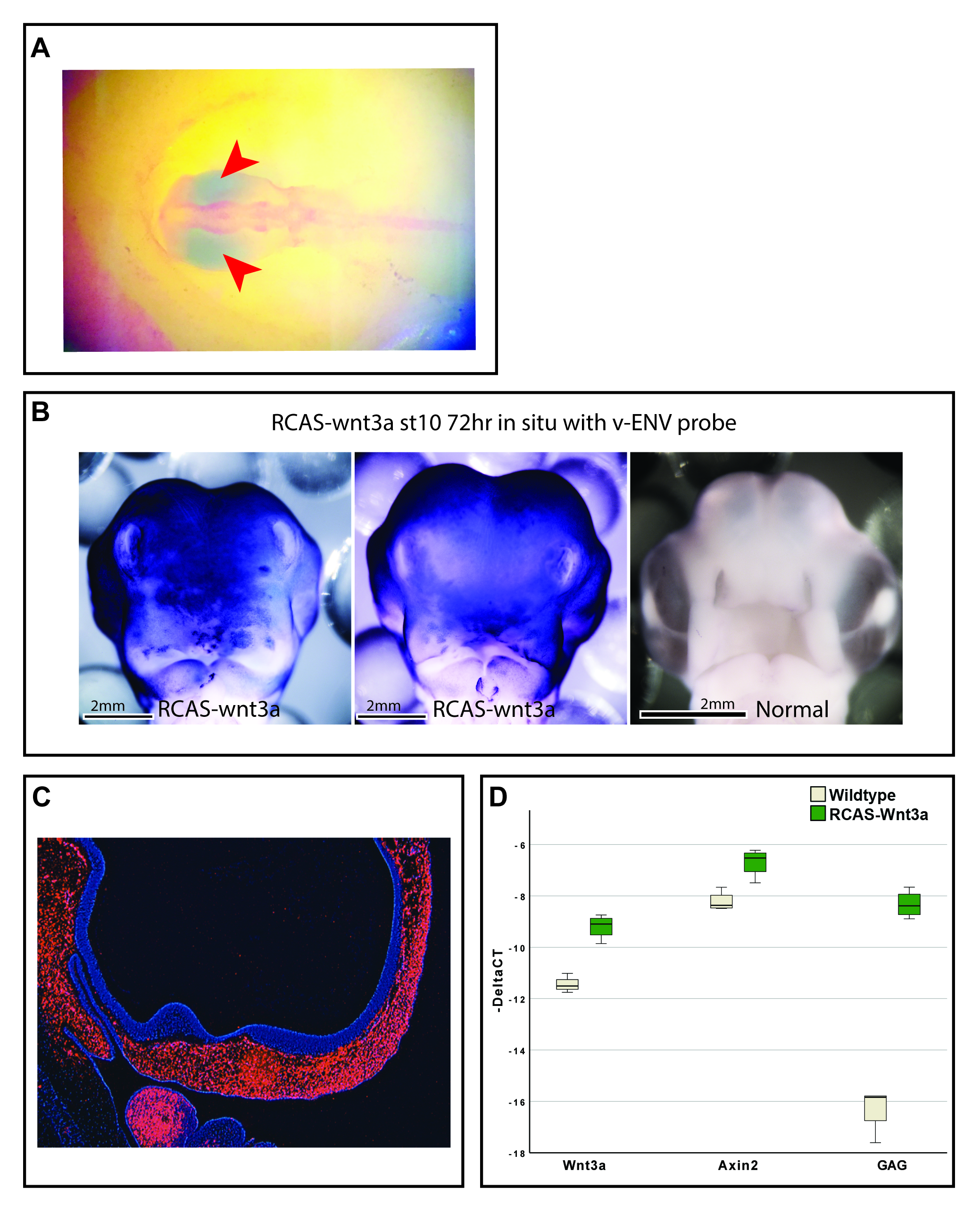

Supplement: Supplementary Figure 1 — Experimental procedure and validation of Wnt3a overexpression. (A) Picture of a St10 chicken indicating the sites of RCAS injection (red arrows). (B) Whole mount in situ hybridization for v-ENV. (C) In situ hybridization in section for v-ENV in red showing that the RCAS infection is mesenchyme specific, blue nuclear staining. (D) Boxplot representing differences -delta CT for WNT3A, AXIN2, and GAG expression. [file Image_1.TIF]

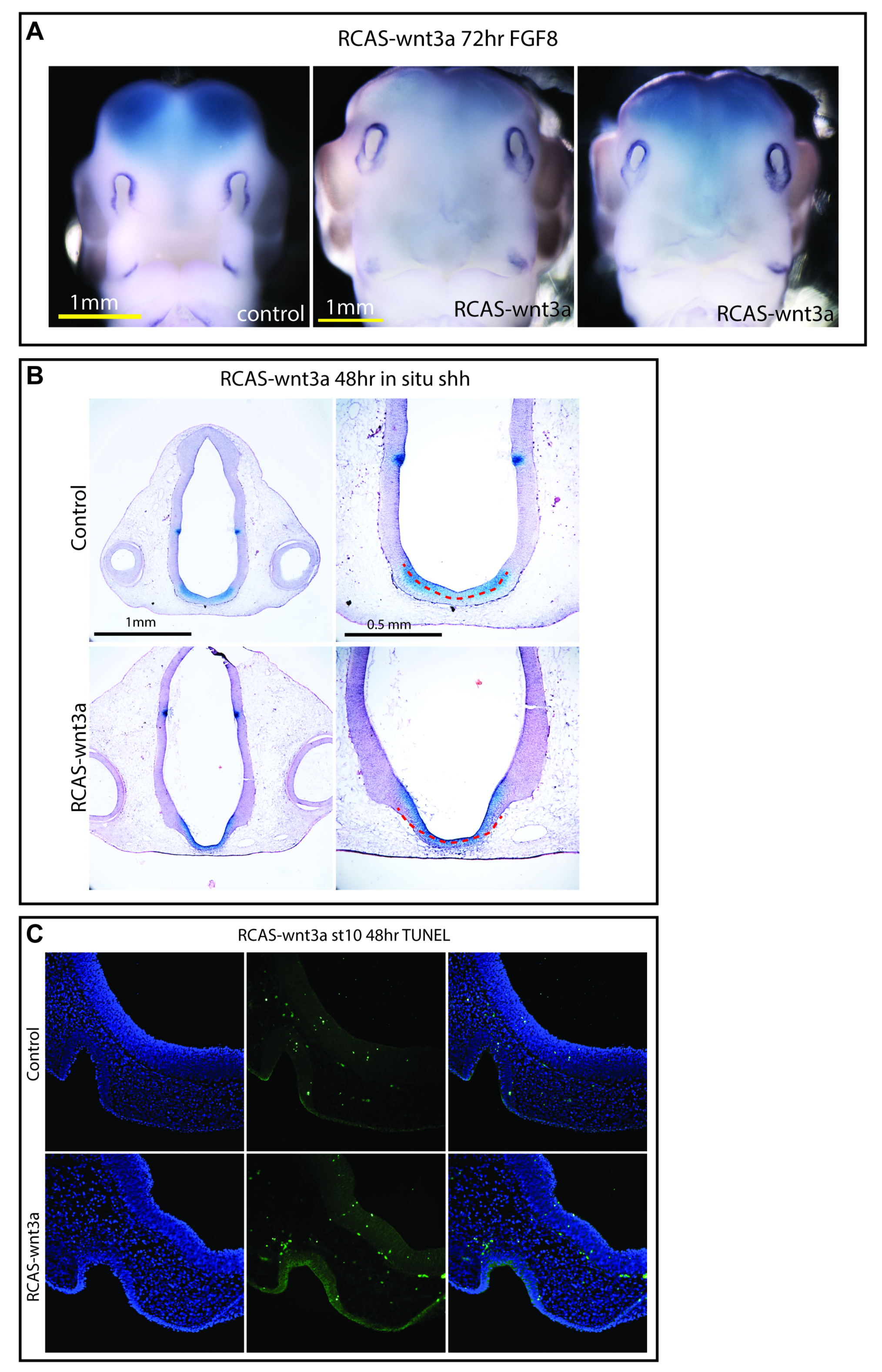

Supplement: Supplementary Figure 2 — Ectopic expression of Wnt3a affects Shh expression but not FGF8 and apoptosis. (A) Whole embryo in situ hybridization for FGF8. (B) In situ hybridization for Shh in head section. (C) TUNEL assay in head section in green cell death in blue nuclear staining. [file Image_2.TIF]

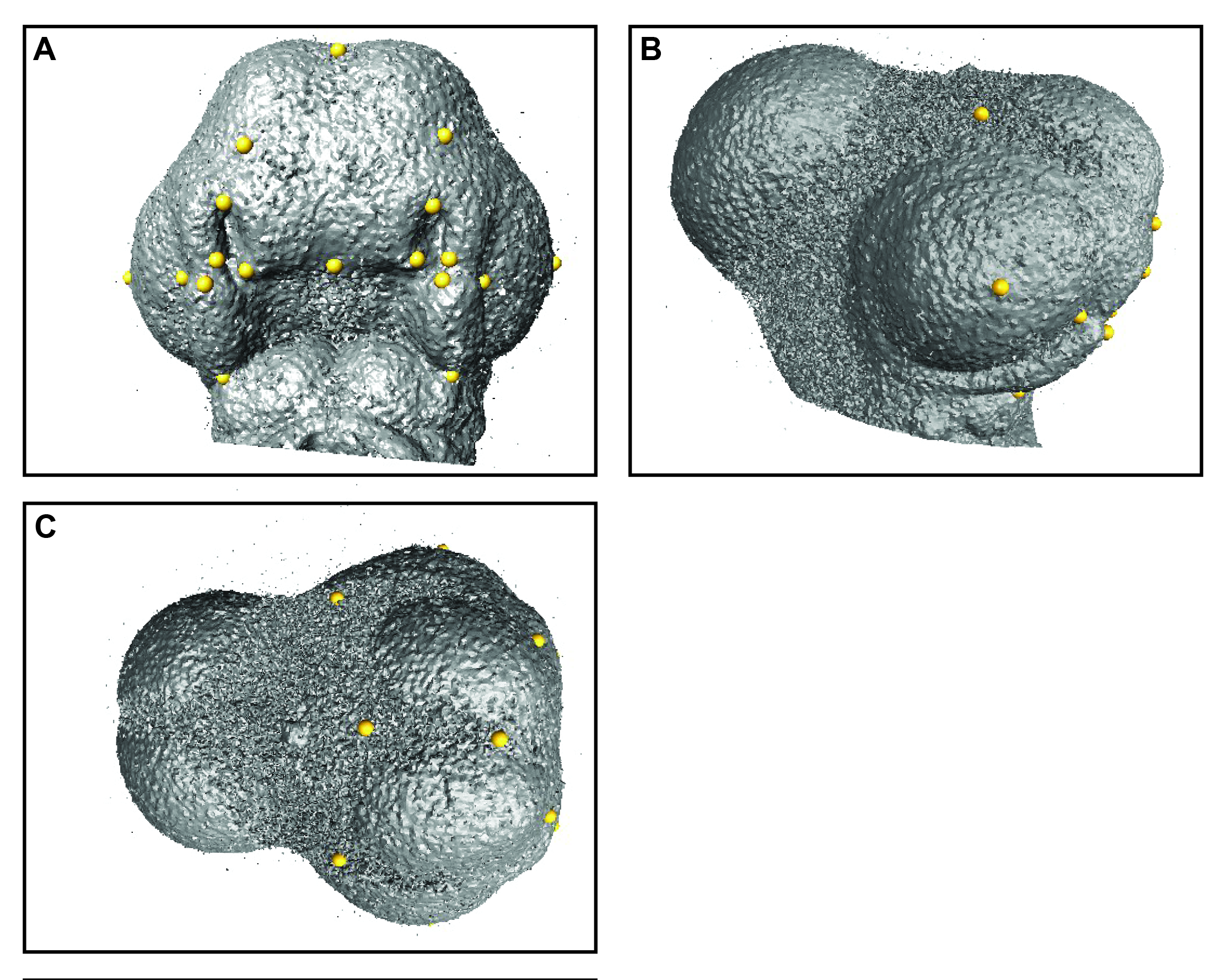

Supplement: Supplementary Figure 3 — Landmarks used to quantify shape of the head. Surface render of the head in gray and 21 landmarks in yellow in anterior (A), lateral (B), and dorsal (C) view. [file Image_3.TIF]

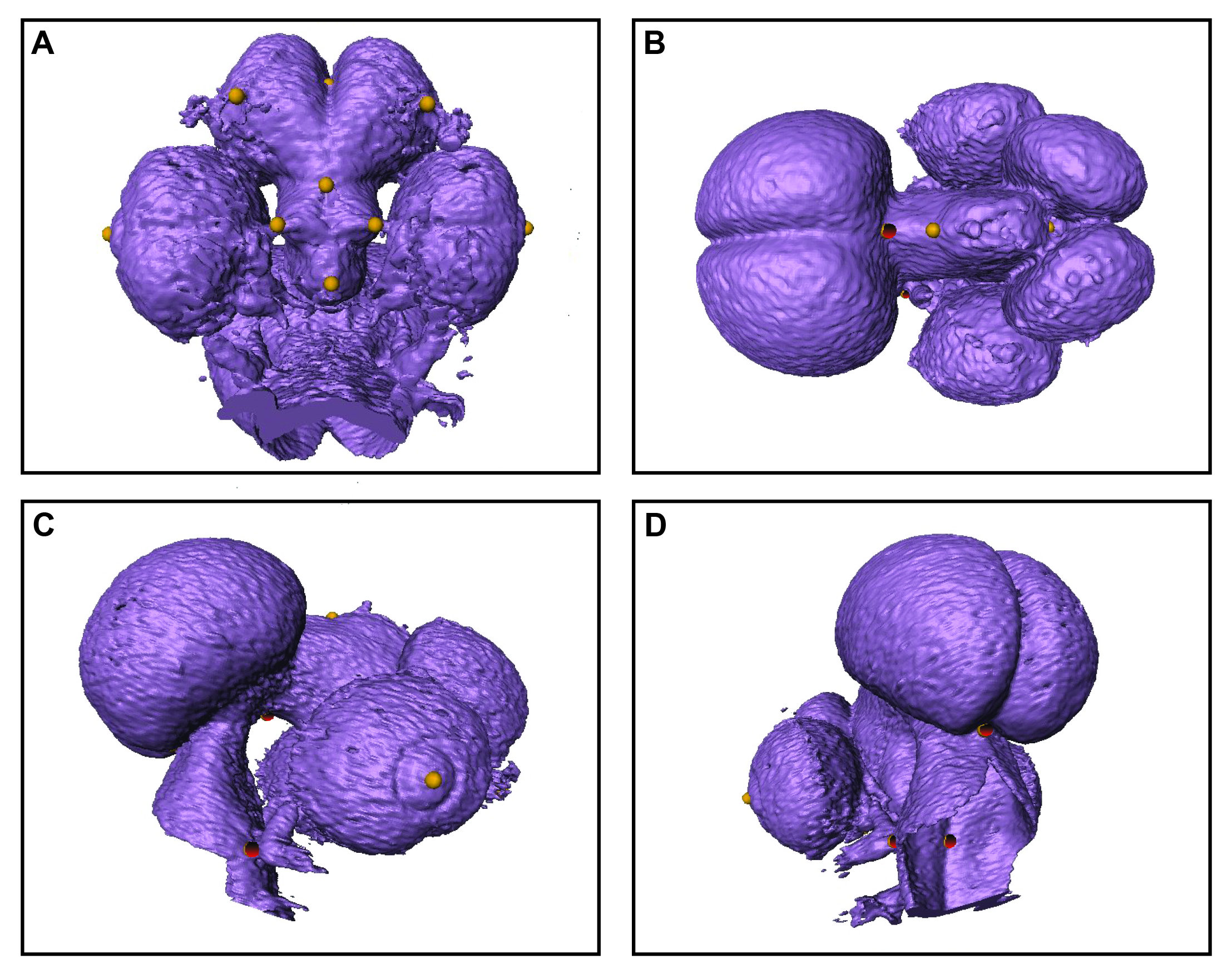

Supplement: Supplementary Figure 4 — Landmarks used to quantify shape of the brain. Surface render of the brain in purple and 17 landmarks in yellow (forebrain) and red (mid-hindbrain) in ventral (A), dorsal (B), lateral (C), and posterior oblique (D) view. [file Image_4.TIF]

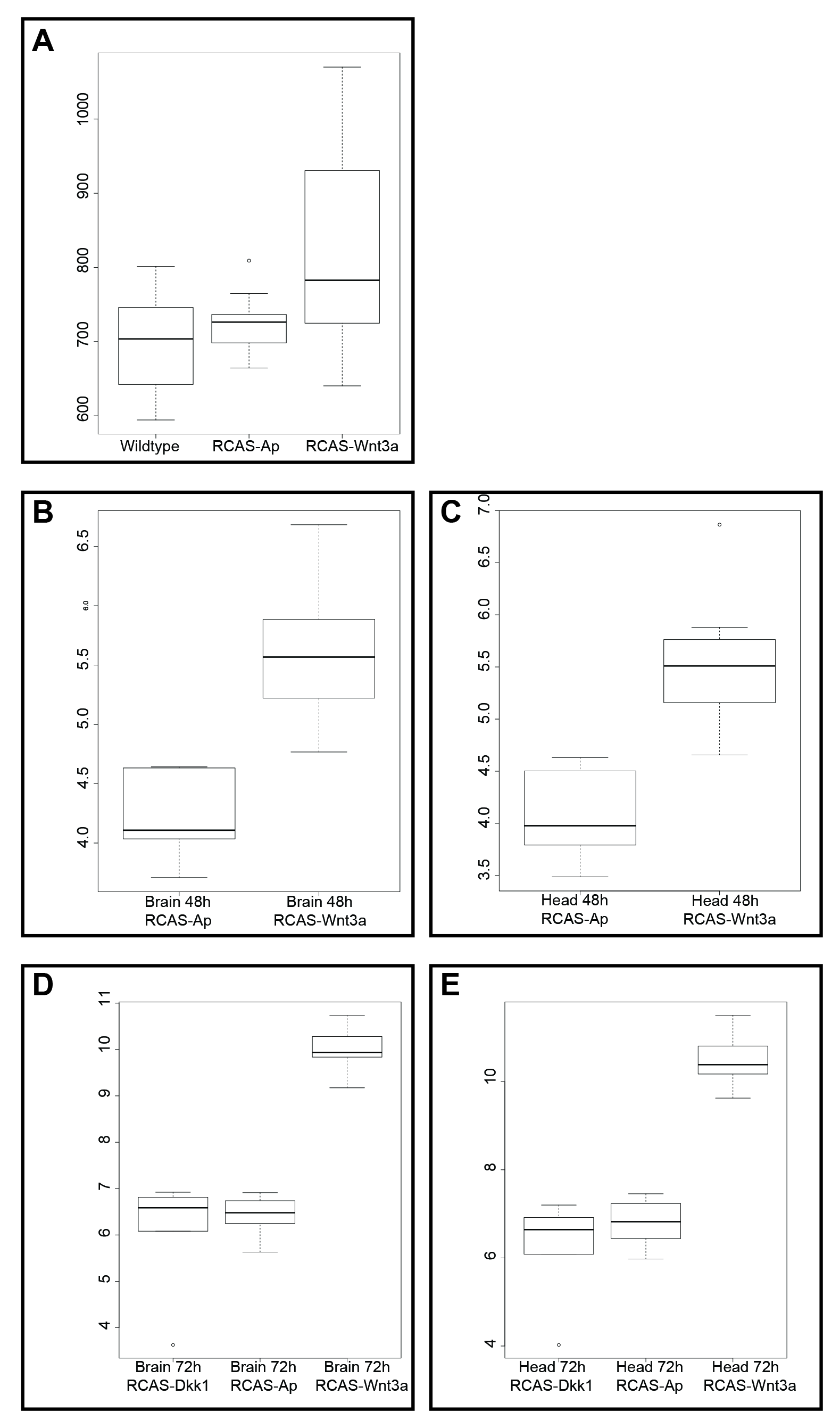

Supplement: Supplementary Figure 5 — Comparison of centroid size across experiments. (A) Centroid size of chicken head at HH22 used for the FEZ analysis. (B) Centroid size of the brain of chickens at HH18 based on 17 landmarks. (C) Centroid size of the head of chickens at HH18 based on 21 landmarks. (D) Centroid size of the brain of chickens at HH22 based on 17 landmarks. (E) Centroid size of the head of chickens at HH22 based on 21 landmarks. [file Image_5.TIF]
