# Supplementary material for: Wnt Signaling Drives Correlated Changes in Facial Morphology and Brain Shape
Source: Front Cell Dev Biol. 2021 Mar 29;9:644099. doi: 10.3389/fcell.2021.644099 (PMC8039397; doi:10.3389/fcell.2021.644099)
Supplement: Supplementary Table 1 — qPCR data of GAPDH, WNT3A, AXIN2, and GAG expression in wild-types and RCAS-Wnt3a. [file Table_1.DOCX]

Table S1

qPCR data of *GAPDH*, *WNT3A*, *AXIN2* and *GAG* expression in wildtypes and RCAS-Wnt3a.

| Gene | Sample | Ct |
| --- | --- | --- |
| GAPDH | control1 | 18,26 |
| GAPDH | control1 | 17,95 |
| wnt3a | control1 | 29,69 |
| wnt3a | control1 | 30,03 |
| Axin2 | control1 | 25,89 |
| Axin2 | control1 | 25,65 |
| GAG | control1 | 36,08 |
| GAG | control1 | 35,35 |
|  |  |  |
| GAPDH | control2 | 18,45 |
| GAPDH | control2 | 18,25 |
| wnt3a | control2 | 29,78 |
| wnt3a | control2 | 29,96 |
| Axin2 | control2 | 26,9 |
| Axin2 | control2 | 26,78 |
| GAG | control2 | 34,25 |
| GAG | control2 | 34,03 |
|  |  |  |
| GAPDH | control3 | 18,51 |
| GAPDH | control3 | 18,39 |
| wnt3a | control3 | 29,96 |
| wnt3a | control3 | 29,94 |
| Axin2 | control3 | 26,95 |
| Axin2 | control3 | 26,85 |
| GAG | control3 | 34,42 |
| GAG | control3 | 34,27 |
|  |  |  |
| GAPDH | control4 | 18,82 |
| GAPDH | control4 | 18,92 |
| wnt3a | control4 | 29,83 |
| wnt3a | control4 | 29,94 |
| Axin2 | control4 | 27,15 |
| Axin2 | control4 | 27,16 |
| GAG | control4 | 34,62 |
| GAG | control4 | 34,7 |

| Gene | Sample | Ct |
| --- | --- | --- |
| GAPDH | RCAS-wnt3a 3 | 16,33 |
| GAPDH | RCAS-wnt3a 3 | 16,54 |
| wnt3a | RCAS-wnt3a 3 | 25,48 |
| wnt3a | RCAS-wnt3a 3 | 25,4 |
| Axin2 | RCAS-wnt3a 3 | 22,81 |
| Axin2 | RCAS-wnt3a 3 | 22,92 |
| GAG | RCAS-wnt3a 3 | 25,47 |
| GAG | RCAS-wnt3a 3 | 25,19 |
|  |  |  |
| GAPDH | RCAS-wnt3a 4 | 17,04 |
| GAPDH | RCAS-wnt3a 4 | 17,25 |
| wnt3a | RCAS-wnt3a 4 | 26,28 |
| wnt3a | RCAS-wnt3a 4 | 26,37 |
| Axin2 | RCAS-wnt3a 4 | 23,74 |
| Axin2 | RCAS-wnt3a 4 | 23,79 |
| GAG | RCAS-wnt3a 4 | 25,64 |
| GAG | RCAS-wnt3a 4 | 25,78 |
|  |  |  |
| GAPDH | RCAS-wnt3a 5 | 16,42 |
| GAPDH | RCAS-wnt3a 5 | 16,4 |
| wnt3a | RCAS-wnt3a 5 | 26,25 |
| wnt3a | RCAS-wnt3a 5 | 26,28 |
| Axin2 | RCAS-wnt3a 5 | 23,87 |
| Axin2 | RCAS-wnt3a 5 | 23,93 |
| GAG | RCAS-wnt3a 5 | 24,59 |
| GAG | RCAS-wnt3a 5 | 24,65 |
|  |  |  |
| GAPDH | RCAS-wnt3a 6 | 17,75 |
| GAPDH | RCAS-wnt3a 6 | 17,81 |
| wnt3a | RCAS-wnt3a 6 | 26,46 |
| wnt3a | RCAS-wnt3a 6 | 26,59 |
| Axin2 | RCAS-wnt3a 6 | 23,9 |
| Axin2 | RCAS-wnt3a 6 | 24,11 |
| GAG | RCAS-wnt3a 6 | 25,38 |
| GAG | RCAS-wnt3a 6 | 25,5 |
