# Supplementary material for: Wnt Signaling Drives Correlated Changes in Facial Morphology and Brain Shape
Source: Front Cell Dev Biol. 2021 Mar 29;9:644099. doi: 10.3389/fcell.2021.644099 (PMC8039397; doi:10.3389/fcell.2021.644099)
Supplement: Supplementary Table 2 — Quantification of total cells and PHH3 positive cells in mesenchyme and neural ectoderm in sections. [file Table_2.DOCX]

Table S2

Quantification of total cells and PHH3 positive cells in mesenchyme and neural ectoderm in sections.

Mesenchyme

|  | RCAS-wnt3a | total cells | PHH3(+) cells |
| --- | --- | --- | --- |
| Embryo 1 | 1 | 1157 | 47 |
|  | 8 | 982 | 67 |
|  | 9 | 950 | 64 |
| Embryo 2 | 10 | 1167 | 63 |
|  | 11 | 1356 | 62 |
|  | 12 | 998 | 21 |
|  | 13 | 1087 | 61 |
| Embryo 3 | 14 | 1289 | 35 |
|  | 15 | 1098 | 96 |
|  | 16 | 995 | 109 |
|  | 17 | 1423 | 41 |
| Embryo 4 | 18 | 1263 | 61 |
|  | 19 | 1052 | 86 |
|  | 20 | 1189 | 39 |

|  | RCAS-AP | total cells | PHH3(+) cells |
| --- | --- | --- | --- |
| Embryo 1 | 1 | 1150 | 73 |
|  | 1-1. | 1056 | 62 |
|  | 2 | 772 | 44 |
|  | 3 | 1099 | 36 |
|  | 4 | 693 | 61 |
|  | 5 | 659 | 61 |
| Embryo 2 | 6 | 1017 | 41 |
|  | 7 | 1390 | 44 |
|  | 8 | 1293 | 52 |
|  | 9 | 1272 | 64 |
| Embryo 3 | 10 | 1300 | 67 |
|  | 11 | 1350 | 58 |
|  | 12 | 1245 | 51 |
|  | 13 | 1038 | 68 |
|  | 14 | 1274 | 52 |
| Embryo 4 | 15 | 1154 | 63 |
|  | 16 | 1015 | 53 |
|  | 17 | 946 | 65 |
|  | 18 | 998 | 64 |
|  | 19 | 1011 | 58 |

Neural Ectoderm

| RCAS-wnt3a | total cells | PHH3(+) cells |
| --- | --- | --- |
| Embryo 1 | 338 | 26 |
| Embryo 2 | 340 | 25 |
| Embryo 3 | 544 | 32 |
| Embryo 4 | 521 | 35 |

| RCAS-AP | total cells | PHH3(+) cells |
| --- | --- | --- |
| Embryo 1 | 354 | 30 |
| Embryo 2 | 446 | 29 |
| Embryo 3 | 494 | 26 |
| Embryo 4 | 514 | 32 |
