# Supplementary material for: Wnt Signaling Drives Correlated Changes in Facial Morphology and Brain Shape
Source: Front Cell Dev Biol. 2021 Mar 29;9:644099. doi: 10.3389/fcell.2021.644099 (PMC8039397; doi:10.3389/fcell.2021.644099)
Supplement: Supplementary Table 11 — R script used in analyses. [file Table_11.DOCX]

library(rgl)

library(ape)

library(geomorph)

library(Morpho)

library (Rvcg)

##importing files

landmarks = read.table("landmarksW.txt", header=T, row.names=1)

groups = read.table("groupsW.txt", header=T, row.names=1)

lm_array = arrayspecs(landmarks, 21, 3)

FEZ = read.table("FEZ2.txt", header=T, row.names=1)

landmarks48H = read.table("landmarks_48H.txt", header=T, row.names=1)

groups48 = read.table("group_48.txt", header=T, row.names=1)

lm_array48H = arrayspecs(landmarks48H, 21, 3)

landmarks48B = read.table("landmarks_48_BR.txt", header=T, row.names=1)

lm_array48B = arrayspecs(landmarks48B, 17, 3)

landmarks72H = read.table("landmarks_72H.txt", header=T, row.names=1)

groups72 = read.table("group_72-BH.txt", header=T, row.names=1)

lm_array72H = arrayspecs(landmarks72H, 21, 3)

landmarks72B = read.table("landmarks_72B.txt", header=T, row.names=1)

lm_array72B = arrayspecs(landmarks72B, 17, 3)

##general procrustes alignment

gpa = gpagen(lm_array)

gpa48H = gpagen(lm_array48H)

gpa48B = gpagen(lm_array48B)

gpa72H = gpagen(lm_array72H)

gpa72B = gpagen(lm_array72B)

##extract centroid size

gpa.csize = gpa$Csize

boxplot(gpa.csize~as.matrix(groups[,1]))

gpa.csize48H = gpa48H$Csize

boxplot(gpa.csize48H~as.matrix(groups48[,1]))

gpa.csize48B = gpa48B$Csize

boxplot(gpa.csize48B~as.matrix(groups48[,1]))

plot (gpa.csize48H, gpa.csize48B, pch =19, col = groups48$group, cex = 1.5)

gpa.csize72H = gpa72H$Csize

boxplot(gpa.csize72H~as.matrix(groups72[,1]))

gpa.csize72B = gpa72B$Csize

boxplot(gpa.csize72B~as.matrix(groups72[,1]))

plot (gpa.csize72H, gpa.csize72B, pch =19, col = groups72$group, cex = 1.5)

write.table(gpa.csize48H, "csize48H.txt", sep="\t")

##save median shape as a reference

ref = mshape(gpa$coords)

##principal coordinates analysis

PCA = plotTangentSpace(gpa$coords, warpgrids=TRUE, groups=(groups$group), label=T)

PCA48H = plotTangentSpace(gpa48H$coords, warpgrids=TRUE, groups=(groups48$group), label=T)

summary (PCA48H)

PCA48B = plotTangentSpace(gpa48B$coords, warpgrids=TRUE, groups=(groups48$group), label=T)

summary (PCA48B)

PCA72H = plotTangentSpace(gpa72H$coords, warpgrids=TRUE, groups=(groups72$group), label=T)

summary (PCA72H)

PCA72B = plotTangentSpace(gpa72B$coords, warpgrids=TRUE, groups=(groups72$group), label=T)

summary (PCA72B)

## prcomp in R, you can standardize your variables using scale = TRUE scale ; all variables to have a variance (and sd) of 1

pca_FEZ = prcomp(FEZ, scale = TRUE)

summary (pca_FEZ)

plot (pca_FEZ$x[,1], pca_FEZ$x[,2], pch =19, col = groups$group, cex = 1.5)

text (pca_FEZ$x[,1], pca_FEZ$x[,2], adj=1,

labels = row.names (pca_FEZ$x), cex = 0.5)

###extracting data

scores48B = PCA48B$pc.scores

write.table(scores48B, "Scores48B.txt", sep="\t")

scores48H = PCA48H$pc.scores

write.table(scores48H, "Scores48H.txt", sep="\t")

scores72B = PCA72B$pc.scores

write.table(scores72B, "Scores72B.txt", sep="\t")

scores72H = PCA72H$pc.scores

write.table(scores72H, "Scores72H.txt", sep="\t")

scoresW = PCA$pc.scores

write.table(scoresW, "ScoresW.txt", sep="\t")

scoresFEZ = pca_FEZ$x

write.table(scoresFEZ, "FEZ2_pca_Score.txt", sep="\t")

write.table(gpa.csize48H, "Csize48H.txt", sep="\t")

### to find meanshape and to produce meshes for the extreme PCs

MeanShape_tot = findMeanSpec(gpa72B$coords)

mean.mesh <- read.ply("Normal_21_3W_low.ply", addNormals = FALSE)

land_CTRL = read.table("LAND_normal_21-3W.txt", header=T, row.names=1)

lm_arrayCTRL = arrayspecs(land_CTRL, 21, 3)

mean.points <- lm_arrayCTRL[,,2]

ref = mshape(gpa$coords)

mean.refwarp <- warpRefMesh(mean.mesh, mean.points, ref)

PC1_min_Shape <- plotRefToTarget(ref,PCA$pc.shapes$PC1min, mesh = mean.refwarp, method="surface")

PC1_max_Shape <- plotRefToTarget(ref,PCA$pc.shapes$PC1max, mesh = mean.refwarp, method="surface")

PC1_Min_dots <- plotRefToTarget(ref,PCA$pc.shapes$PC1max, method="vector")

PC2_min_Shape <- plotRefToTarget(ref,PCA$pc.shapes$PC2min, mesh = mean.refwarp, method="surface")

PC2_max_Shape <- plotRefToTarget(ref,PCA$pc.shapes$PC2max, mesh = mean.refwarp, method="surface")

###procrustes

gpa72B = gpagen(lm_array72B)

findMeanSpec(gpa72B$coords)

mean.mesh72B <- read.ply("MM_RCAS-DKK1-72_6.ply", addNormals = FALSE)

mean.points72B <- lm_array72B[,,11]

ref72B = mshape(gpa72B$coords)

mean.refwarp72B <- warpRefMesh(mean.mesh72B, mean.points72B, ref72B)

PC1_min_Shape72B <- plotRefToTarget(ref72B,PCA72B$pc.shapes$PC1min, mesh = mean.refwarp72B, method="surface")

PC1_max_Shape72B <- plotRefToTarget(ref72B,PCA72B$pc.shapes$PC1max, mesh = mean.refwarp72B, method="surface")

PC2_min_Shape72B <- plotRefToTarget(ref72B,PCA72B$pc.shapes$PC2min, mesh = mean.refwarp72B, method="surface")

PC2_max_Shape72B <- plotRefToTarget(ref72B,PCA72B$pc.shapes$PC2max, mesh = mean.refwarp72B, method="surface")

mean.mesh72H <- read.ply("MM_RCAS-DKK1-72_5.ply", addNormals = FALSE)

mean.points72H <- lm_array72H[,,10]

ref72H = mshape(gpa72H$coords)

mean.refwarp72H <- warpRefMesh(mean.mesh72H, mean.points72H, ref72H)

PC2_min_Shape72H <- plotRefToTarget(ref72H,PCA72H$pc.shapes$PC2min, mesh = mean.refwarp72H, method="surface")

PC2_max_Shape72H <- plotRefToTarget(ref72H,PCA72H$pc.shapes$PC2max, mesh = mean.refwarp72H, method="surface")

### to find meanshape and to produce meshes for the extreme for 48

findMeanSpec(gpa48B$coords)

mean.mesh48B <- read.ply("WNT3A-48_12.ply", addNormals = FALSE)

mean.points48B <- lm_array48B[,,15]

ref48B = mshape(gpa48B$coords)

mean.refwarp48B <- warpRefMesh(mean.mesh48B, mean.points48B, ref48B)

PC1min_shape_48B <- plotRefToTarget(ref48B,PCA48B$pc.shapes$PC1min, mesh = mean.refwarp48B, method="surface")

PC1max_Shape_48B <- plotRefToTarget(ref48B,PCA48B$pc.shapes$PC1max, mesh = mean.refwarp48B, method="surface")

PC2min_shape_48B <- plotRefToTarget(ref48B,PCA48B$pc.shapes$PC2min, mesh = mean.refwarp48B, method="surface")

PC2max_Shape_48B <- plotRefToTarget(ref48B,PCA48B$pc.shapes$PC2max, mesh = mean.refwarp48B, method="surface")

findMeanSpec(gpa48H$coords)

mean.mesh48H <- read.ply("WNT3-48_9.ply", addNormals = FALSE)

mean.points48H <- lm_array48H[,,13]

ref48H = mshape(gpa48H$coords)

mean.refwarp48H <- warpRefMesh(mean.mesh48H, mean.points48H, ref48H)

PC2min_shape_48H <- plotRefToTarget(ref48H,PCA48H$pc.shapes$PC2min, mesh = mean.refwarp48H, method="surface")

PC2max_Shape_48H <- plotRefToTarget(ref48H,PCA48H$pc.shapes$PC2max, mesh = mean.refwarp48H, method="surface")

findMeanSpec(gpa72B$coords)

## eigenvalues

PCA$pc.summary

## to plot other pc component

plot (PCA$pc.scores [,1], PCA$pc.scores [,2], pch =19, col = groups$group, cex = 1.5)

text (PCA$pc.scores [,1], PCA$pc.scores [,2], adj=1,

labels = row.names (PCA$pc.scores), cex = 0.5)

plot (PCA$pc.scores [,3], PCA$pc.scores [,4], pch =19, col = groups$group, cex = 1.5)

text (PCA$pc.scores [,3], PCA$pc.scores [,4], adj=1,

labels = row.names (PCA$pc.scores), cex = 0.5)

plot (PCA48H$pc.scores [,1], PCA48H$pc.scores [,2], pch =19, col = groups48$group, cex = 1.5)

text (PCA$pc.scores [,1], PCA$pc.scores [,2], adj=1,

labels = row.names (PCA$pc.scores), cex = 0.5)

plot (PCA48B$pc.scores [,1], PCA48B$pc.scores [,2], pch =19, col = groups48$group, cex = 1.5)

text (PCA$pc.scores [,1], PCA$pc.scores [,2], adj=1,

labels = row.names (PCA$pc.scores), cex = 0.5)

plot (data_CCA[,1], data_CCA[,2], pch =19, col = groups$group, cex = 1.5)

text (data_CCA[,1], data_CCA[,2], adj=1,

labels = row.names (data_CCA), cex = 0.5)

plot (PCA$pc.scores [,1], pca_FEZ$x [,1], pch =19, col = groups$group, cex = 1.5)

text (PCA$pc.scores [,1], pca_FEZ$x [,1], adj=1,

labels = row.names (PCA$pc.scores), cex = 0.5)

### compare the same module between different groups

gpa72.hb = gpagen(lm_array72B[c(6,7,8,15,16,17),,])

gpa72.fb = gpagen(lm_array72B[c(1,2,3,4,5,8,9,10,11,12,13,14),,])

gpa.csize72.fb = gpa72.fb$Csize

gpa.csize72.hb = gpa72.hb$Csize

write.table(gpa.csize72.fb, "csize72_fb.txt", sep="\t")

write.table(gpa.csize72.hb, "csize72_hb.txt", sep="\t")

plot (gpa.csize72.fb, gpa.csize72.hb, pch =19, col = groups72$group, cex = 1.5)

boxplot(gpa.csize72.hb~as.matrix(groups72[,1]))

PCA72.hb2 = plotTangentSpace(gpa72.hb$coords, warpgrids=TRUE, groups=(groups72$group), label=T)

summary (PCA72.hb2)

PCA72.fb2 = plotTangentSpace(gpa72.fb$coords, warpgrids=TRUE, groups=(groups72$group), label=T)

summary (PCA72.fb2)

plot (PCA72.fb2$pc.scores [,1], PCA72.hb2$pc.scores [,1], pch =19, col = groups72$group, cex = 1.5)

scores72.hb = PCA72.hb2$pc.scores

write.table(scores72.hb, "Scores72_with8.hb.txt", sep="\t")

scores72.fb = PCA72.fb2$pc.scores

write.table(scores72.fb, "Scores72_with8.fb.txt", sep="\t")

gpa48.hb = gpagen(lm_array48B[c(6,7,8,15,16,17),,])

gpa48.fb = gpagen(lm_array48B[c(1,2,3,4,5,9,10,11,12,13,14),,])

gpa.csize48.fb = gpa48.fb$Csize

gpa.csize48.hb = gpa48.hb$Csize

plot (gpa.csize48.fb, gpa.csize48.hb, pch =19, col = groups48$group, cex = 1.5)

boxplot(gpa.csize48.hb~as.matrix(groups48[,1]))

write.table(gpa.csize48.fb, "csize48_fb.txt", sep="\t")

write.table(gpa.csize48.hb, "csize48_hb.txt", sep="\t")

PCA48.hb2 = plotTangentSpace(gpa48.hb$coords, warpgrids=TRUE, groups=(groups48$group), label=T)

summary (PCA48.hb2)

PCA48.fb2 = plotTangentSpace(gpa48.fb$coords, warpgrids=TRUE, groups=(groups48$group), label=T)

summary (PCA48.fb2)

plot (PCA48.fb2$pc.scores [,1], PCA48.hb2$pc.scores [,1], pch =19, col = groups48$group, cex = 1.5)

scores48.hb = PCA48.hb2$pc.scores

write.table(scores48.hb, "Scores48_with8.hb.txt", sep="\t")

scores48.fb = PCA48.fb2$pc.scores

write.table(scores48.fb, "Scores48_with8.fb.txt", sep="\t")

#### if using the procrustes

B72.hb = gpa72B$coords[c(6,7,15,16,17),,]

B72.fb = gpa72B$coords[c(1,2,3,4,5,8,9,10,11,12,13,14),,]

PCA72.hb = plotTangentSpace(B72.hb, warpgrids=TRUE, groups=(groups72$group), label=T)

summary (PCA72.hb)

PCA72.fb = plotTangentSpace(B72.fb, warpgrids=TRUE, groups=(groups72$group), label=T)

summary (PCA72.fb)

plot (PCA72.fb$pc.scores [,1], PCA72.hb$pc.scores [,1], pch =19, col = groups72$group, cex = 1.5)

######## modularity test

land.gps = c("A","A","A","A","A","B","B","B","A","A","A","A","A","A","B","B","B")

TEST_MOD = modularity.test(gpa72B$coords, land.gps, iter=999)

summary(TEST_MOD)

plot(TEST_MOD)

TEST_MOD_w = modularity.test(gpa72B$coords[,,groups72=="W"], land.gps, iter=999)

summary(TEST_MOD_w)

plot(TEST_MOD_w)

TEST_MOD_ic = modularity.test(gpa72B$coords[,,groups72=="IC"], land.gps, iter=999)

summary(TEST_MOD_ic)

plot(TEST_MOD_ic)

TEST_MOD_d = modularity.test(gpa72B$coords[,,groups72=="D"], land.gps, iter=999)

summary(TEST_MOD_d)

plot(TEST_MOD_d)

TEST_MOD48 = modularity.test(gpa48B$coords, land.gps, iter=999)

summary(TEST_MOD48)

plot(TEST_MOD48)

TEST_MOD48w = modularity.test(gpa48B$coords[,,groups48=="W"], land.gps, iter=999)

summary(TEST_MOD48w)

plot(TEST_MOD48w)

TEST_MOD48ic = modularity.test(gpa48B$coords[,,groups48=="IC"], land.gps, iter=999)

summary(TEST_MOD48ic)

plot(TEST_MOD48ic)

##extracting scores of PCA on samples

scoresW = PCA$pc.scores

scoresFEZ = pca_FEZ$x

write.table(scoresFEZ, "FEZ2_pca_Score.txt", sep="\t")

write.table(scoresW, "ScoresW.txt", sep="\t")

##reformat centroid size

csizeW =as.matrix(gpa.csize)

csizeMA =as.matrix(gpaMA.csize)

FEZw =as.matrix(FEZ)

#### non parametric test

library (dunn.test)

NPtestHEAD = dunn.test (x = scoresW[,1]+scoresW[,2]+scoresW[,3]+scoresW[,4]+scoresW[,5], g = groups[,1], method = "bonferroni")

scoresFEZ = pca_FEZ$x

NPtest48hb = dunn.test (x = scoresFEZ[,1]+scoresFEZ[,2]+scoresFEZ[,3]+scoresFEZ[,4], g = groups[,1], method = "bonferroni")

NPtestFEZcs = dunn.test (x = csizeW, g = groups[,1], method = "bonferroni")

NPtestFEZarea = dunn.test (x = FEZ[,3], g = groups[,1], method = "bonferroni")

NPtestFEZslope = dunn.test (x = FEZ[,2], g = groups[,1], method = "bonferroni")

NPtestFEZslope = dunn.test (x = FEZ[,2], g = groups[,1], method = "bonferroni")

NPtestFEZcom = dunn.test (x = FEZ[,4], g = groups[,1], method = "bonferroni")

scores72h = PCA72H$pc.scores

NPtest72H = dunn.test (x = scores72h[,1]+scores72h[,2]+scores72h[,3], g = groups72[,1], method = "bonferroni")

scores72B = PCA72B$pc.scores

NPtest72B = dunn.test (x = scores72B[,1]+scores72B[,2]+scores72B[,3], g = groups72[,1], method = "bonferroni")

scores48h = PCA48H$pc.scores

NPtest48H = dunn.test (x = scores48h[,1]+scores48h[,2]+scores48h[,3]+scores48h[,4]+scores48h[,5], g = groups48[,1], method = "bonferroni")

scores48B = PCA48B$pc.scores

NPtest48B = dunn.test (x = scores48B[,1]+scores48B[,2]+scores48B[,3]+scores48B[,4], g = groups48[,1], method = "bonferroni")

scores72_fb = PCA72.fb2$pc.scores

NPtest72fb = dunn.test (x = scores72_fb[,1]+scores72_fb[,2]+scores72_fb[,3]+scores72_fb[,4]+scores72_fb[,5], g = groups72[,1], method = "bonferroni")

scores72_hb = PCA72.hb2$pc.scores

NPtest72hb = dunn.test (x = scores72_hb[,1]+scores72_hb[,2]+scores72_hb[,3]+scores72_hb[,4]+scores72_hb[,5], g = groups72[,1], method = "bonferroni")

scores48_fb = PCA48.fb2$pc.scores

NPtest48fb = dunn.test (x = scores48_fb[,1]+scores48_fb[,2]+scores48_fb[,3]+scores48_fb[,4]+scores48_fb[,5], g = groups48[,1])

scores48_hb = PCA48.hb2$pc.scores

NPtest48hb = dunn.test (x = scores48_hb[,1]+scores48_hb[,2]+scores48_hb[,3]+scores48_hb[,4]+scores48_hb[,5], g = groups48[,1])
